# Influence of Phosphorus Structures and Their Oxidation States on Flame-Retardant Properties of Polyhydroxyurethanes

**DOI:** 10.3390/molecules28020611

**Published:** 2023-01-06

**Authors:** Maxinne Denis, Guilhem Coste, Rodolphe Sonnier, Sylvain Caillol, Claire Negrell

**Affiliations:** 1 ICGM, Université de Montpellier, CNRS, ENSCM, 34000 Montpellier, France; 2 Polymers Composites and Hybrids (PCH), IMT Mines Ales, 30100 Ales, France

**Keywords:** non-isocyanate polyurethane, polyhydroxyurethane, flame retardant, phosphinate, phosphonate

## Abstract

This article focuses on the synthesis of polyhydroxyurethane (PHU) materials containing novel phosphorus flame retardants (FR). Four different phosphorus compounds were grafted onto cyclic carbonate: 9,10-dihydro-9-oxa-10-phosphaphenanthrene-10-oxide (DOPO), diethyl phosphite (DEP), diphenyl phosphite (DPP) and dibenzo[d,f][1,3,2]dioxaphosphepine 6-oxide (BPPO). Thus, three novel phosphorus reactive cyclic carbonates which have never been reported so far were synthetized. Phosphorus FR containing PHU materials were characterized by FTIR to evidence the total conversion of the cyclic carbonate. Moreover, the gel contents up to 80% confirmed the formation of the polymer network. Then, the thermal stability and the flame-retardant properties were investigated by thermogravimetric analyses, cone calorimeter and pyrolysis combustion flow calorimeter. The mode of action of phosphorus compounds, depending on the oxidation state, was especially highlighted. Phosphonate (+III) provided better action in a condensed phase than phosphinate thanks to a more efficient char formation. Among phosphonates, differences were observed in terms of char-formation rate and expansion. DEP provided the best flame-retardant properties, with a reduction of 76% of pHRR with 2 wt% of phosphorus in cone calorimeter analysis. Therefore, this article highlighted the different modes of action of phosphorus flame retardants, depending on the oxidation state of phosphorus, in PHU materials.

## 1. Introduction

Polyurethane (PU) is one of the most commonly used polymers in industry. Indeed, its global production in 2020 was nearly 25 Mt, ranking 5th among all polymers, with a global market valued at $71 billion [1,2,3]. PUs are synthetized by the polyaddition reaction of a polyol and a diisocyanate [4]. They are composed of hard and soft segments, which confer their unique properties and structuring [5,6]. PUs are used in many applications such as foams, coatings, elastomers, insulators, sealants, adhesives, medical implants and devices, textiles, etc. [7,8].

As with all organic polymers, PUs have a high content of carbon and hydrogen which are flammable elements, and could release harmful compounds and toxic fumes during combustion. Indeed, when burnt, PUs release high amounts of HCN, HNCO, CO, NO and NO_2_ concentrations and other dangerous gases [9]. Hence, the flammability of PUs considerably limits their use in areas where people’s lives and property are threatened such as construction, transportation, aviation, electrical and electronic equipment. Thus, high flame-retardancy properties are required and these properties are provided by the introduction of a flame retardant (FR) in order to protect consumers during use and reduce the risk associated with polymers flammability [10,11].

Usually, to impart flame-retardant properties to PUs, FR additives are added during the formulation step. Various FR additives such as expandable graphite, organoclay, silica compounds, boric compounds, phosphorus compounds (aluminum phosphate, ammonium polyphosphate, triethyl phosphate), melamine and derivatives have been widely used to provide FR properties to PUs [9,11,12,13,14,15,16,17,18]. However, FR additives have some drawbacks, such as the modification of mechanical and physical properties of formulated polymers. Moreover, since they are not covalently bonded to the network, FR additives can migrate to the surface, leach out and cause toxicity issues [19]. Moreover, due to such migration, the FR properties are lost over the years [20].

The use of reactive halogenated compounds is not an attractive solution; although, they can be covalently bonded to the polymer matrix. Indeed, during combustion, such halogenated compounds release highly toxic, corrosive and potentially carcinogenic degradation products [20,21].

Thus, phosphorus-based reactive FRs are the most promising route to protect polymers from combustion. Wazarkar et al. have reported the synthesis of a phosphate-containing triol used as a reactive FR to prepare PUs coatings [22]. Patel et al. have investigated the synthesis of a phosphate-based flame retardant with gallic acid as a crosslinking agent for PU coatings [23]. Mestry et al. studied the flame-retardant properties of PU coatings synthesized with cardanol-derived phosphate and silicone precursors able to react with isocyanates [24]. Shao et al. have compared the FR properties of a phosphonate and a phosphate-containing aziridinyl groups which could react with carbonyl groups of a previously prepared PU [25]. The results have demonstrated that phosphate FR exhibited higher char yield and thermal stability than phosphonate FR. Various biobased polyols containing phosphorus and nitrogen and derived from castor oil were synthetized by Ding et al. and copolymerized for the synthesis of PUs for FR sealant application [26,27]. In the first article, a phosphate-nitrogen diol was reacted with a diisocyanate; whereas, in the second one, the authors synthetized a PU by reacting diisocyanate, phosphonate-nitrogen diol and phosphinate-nitrogen diol. In both studies, the high char content was explained by the synergistic effect of phosphorus and nitrogen. Bhoyate et al. investigated the FR properties of a novel reactive flame-retardant diol based on diethyl allylphosphonate and used in the synthesis of PU foam (PUF) formulations [28]. This phosphonate-containing diol promoted char formation during combustion. Moreover, most of the studies dealt with the synthesis of novel polyol structures containing phosphinate 9,10-dihydro-9-oxa-10-phosphaphenanthrene 10-oxide (DOPO) as a reactive flame retardant for PU foams, materials or coatings [29,30,31,32].

Nowadays, major concerns are related to the environment and human health. This is why a growing interest in the replacement of toxic and carcinogenic, mutagenic and reprotoxic (CMR) substances, has emerged. Indeed, some of the most common diisocyanates such as methylene diphenyl 4,4′-diisocyanate (MDI) and toluene diisocyanate (TDI), are harmful and recent regulations tend to replace them with the development of new synthetic routes [33,34]. Therefore, Non-Isocyanate PolyUrethanes (NIPUs) have been extensively studied over the past decade [33,35,36,37]. The most promising route to obtain NIPUs is the synthesis of polyhydroxyurethanes (PHUs), obtained by the aminolysis reaction of cyclic carbonates [38,39,40,41,42]. However, only a few cyclic carbonates are commercially available and most of them are very expensive. Fortunately, cyclic carbonates can be easily synthetized by a carbon dioxide carbonation of epoxides. This route also has the advantage to valorize carbon dioxide which is a renewable and cheap resource [7,8,43,44]. Moreover, the presence of urethane linkages is interesting as they promote hydrogen bondings between polymer chains which could improve the physical properties of the final materials, such as adhesion and rigidity.

A previous study focused on different structures of cyclic carbonates functionalized with DOPO in order to obtain PHU foams with FR properties [45]. The best results were obtained with the foam containing 2 wt% of phosphorus and a cyclic carbonate containing two aromatic rings.

**Scheme 1:**
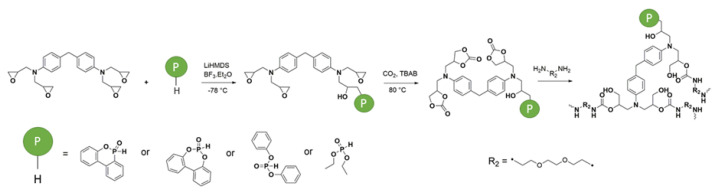
General scheme of the synthesis and polymerization of reactive phosphorus-based FRs.

Hence, cyclic carbonates with two aromatic rings have been selected for the present study. This study investigates the effectiveness of different structures of phosphorylated compounds as well as different oxidation states of phosphorus on the FR properties. Scheme 1 presents the general synthesis of phosphinate-carbonate and phosphonate-carbonates used for the synthesis of PHU materials. Then, the study focused on the comparison of the thermal behavior of phosphinate and phosphonate compounds with similar structures. Lastly, the flame retardancy of different structures of phosphonate and phosphinate compounds was examined.

## 2. Results and Discussion

### 2.1. Synthesis of Monomers and Materials

Before the synthesis of the monomers and PHUs, a complete characterization of raw materials has been carried out in order to determine their functionalities by ^1^H NMR. The EEW calculated with Equation (1) for 4,4′-methylenebis(N,N-diglycidylaniline) (MBDA) was 4.0. The functionalization of one epoxy with phosphorus compound then led tothe functionality of 3.0. MBDA has been chosen for its structure that contains aromatic rings, which would provide char formation during combustion. Moreover, in a previous study, MBDAC exhibited better results than other epoxies due to its two aromatic rings [45]. Different phosphorus contents were introduced in the PHUs, from 0 to 2 wt% phosphorus. The titration of the Jeffamine EDR-148 (EDR-148) confirmed the functionality of two amines. Indeed, the AEW is about 74 g/eq which is half of the molar mass of the amine.

#### 2.1.1. Synthesis of Phosphorylated Cyclic Carbonate MBDAC

The synthesis of phosphorylated carbonate MBDAC occurred in two steps. The first one was the phosphorylation of one epoxy of MBDA and the second one is the carbonation of the remaining epoxies.

Different phosphorus compounds, with a P-H bond, and MBDA were added in a stoichiometric ratio 1:1. The synthesis mechanism of MBDA-DOPO was described in a previous work [45]. This reaction was based on a nucleophilic attack of P^−^, obtained at a high temperature (155 °C), which enabled the epoxide group to open and form a P-C bond as well as a hydroxy group.

**Scheme 2:**
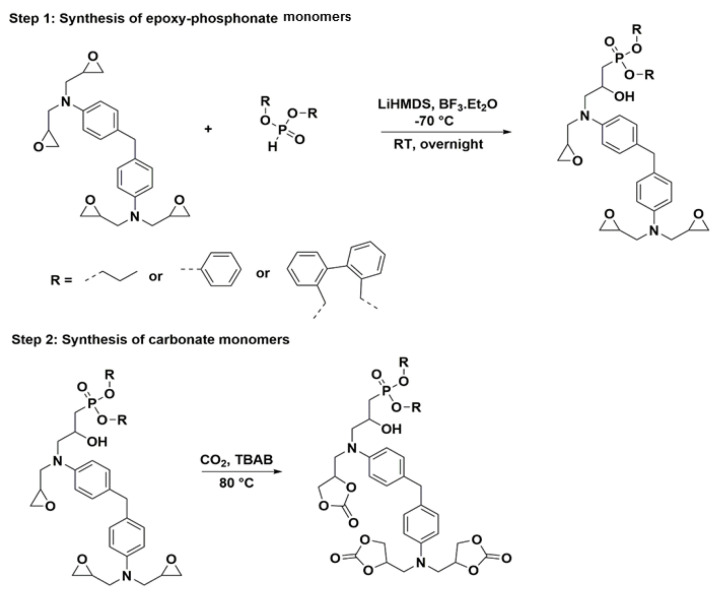
Synthesis of phosphorylated flame-retardant monomers.

The synthesis mechanism of MBDA-phosphonate was also based on a nucleophilic attack of P^−^. Nevertheless, the high temperature was not sufficient to form a P^−^. That is why BF_3_.Et_2_O was added in order to polarize the P-H bond and then facilitate the deprotonation with a strong base such as LiHMDS. The synthesis of MBDA-phosphonate monomers is presented in Scheme 2.

Moreover, the nucleophilic attack occurred on the less substituted carbon of the epoxide leading to the corresponding β-hydroxyphosphinates and β-hydroxyphosphonates. The formation of P-C bond was investigated by ^31^P NMR. Indeed, on the ^31^P NMR spectrum, the disappearance of the chemical shift of P-H bond (between 16 and 0 ppm, depending on the structure of the phosphorus monomers used) was observed. Then, the appearance of the chemical shift at 36 ppm for MBDA-DOPO, 23.9 ppm for MBDA-DPP, 28.8 ppm for MBDA-DEP and 38.6 ppm for MBDA-BPPO is characteristic of the formation of a P-C bond. The ^1^H and ^31^P NMR spectra are presented in supporting information (Figures S7, S8, S19, S20, S25, S26, S13 and S14 for MBDA-DOPO, MBDA-DEP, MBDA-DPP, MBDA-BPPO, respectively). Moreover, the EEW was determined with ^1^H NMR analyses and all the measurements exhibited a value around 3, which confirmed that only one epoxy group per molecule in average has been phosphorylated.

**Figure 1:**
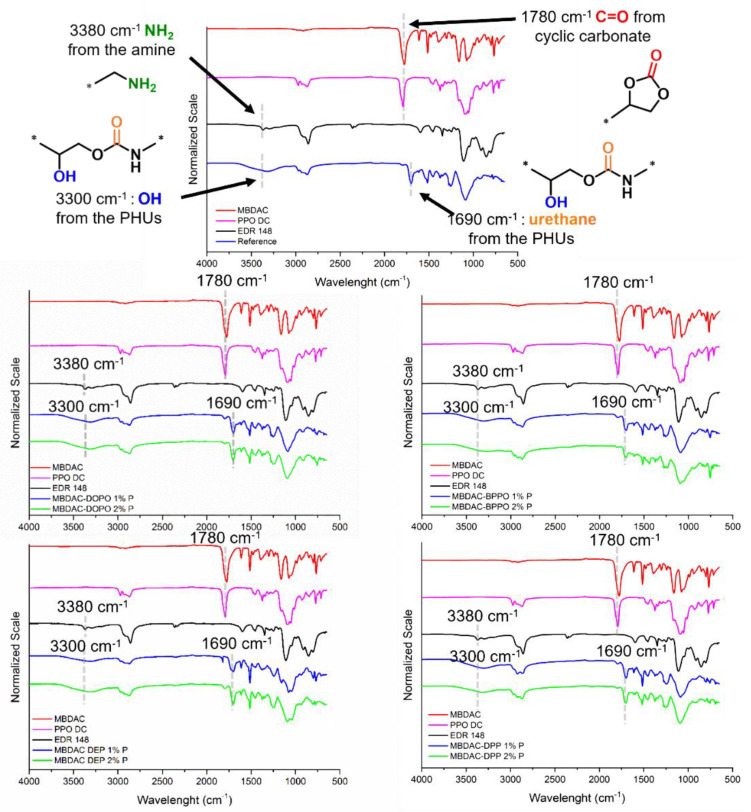
FTIR spectra of the different formulations.

The epoxy monomers obtained during the previous step were carbonated in order to obtain cyclic carbonates as presented in Scheme 2. Such a reaction takes usually place in a pressurized reactor containing a phase transfer catalyst (TBAB), 20 bars of CO_2_ in DCM.52 After 120 h at 80 °C the reaction was stopped and the TBAB was removed by liquid–liquid extraction. The dried products were characterized by ^1^H and ^31^P NMR and FTIR (NMR: Figures S2, S4, S9, S10, S15, S16, S21, S22, S27 and S28 for PPO DC, MBDAC, MBDAC-DOPO, MBDAC-DEP, MBDAC-DPP, MBDAC-BPPO, respectively, and FTIR Figure 1). The ^1^H NMR analysis and Equation (3) were used to determine the CEW of the molecules, which all exhibited a value of 3.

#### 2.1.2. Formulations of Phosphorylated-PHU Materials

In this study, nine different PHU formulations have been studied. They were prepared at 80 °C by the reaction between the different phosphorylated carbonates and EDR 148. The catalyst has been chosen to further previous work in the literature [46]. A 1:1 ratio amine:cyclic carbonate/phosphorylated cyclic carbonate is used in the formulations. Before any formulation, all the solids have been turned into fine powder. First, the carbonate and the phosphorylated carbonate have been mixed 1 min with PPO DC and the thiourea catalyst. Then, the amine was introduced and the formulation was mixed 30 additional seconds.

The crosslinking degree was determined by the measurement of the swelling rate and insoluble content. Finally, thermal properties were studied by DSC and TGA. To determine the influence of the structures of phosphorus-containing compounds on the material combustion behavior, cone calorimeter and PCFC were used.

1. Structural analysis

FTIR analyses were performed to first characterize the monomers. The cyclic carbonate stretching was identified at 1780 cm^−1^. The amine stretching was observed around 3380 cm^−1^. After completion of the reaction of the amine with the MBDAC and PPO DC, the signal assigned to the cyclic carbonate disappeared and that of the C=O from the urethane appeared. The hydroxyl group from the PHU is also observed around 3300 cm^−1^. Figure 1 shows the different FTIR spectra for all the materials and evidences the vanishing of the C=O in the cyclic carbonates on all the thermosets.

To confirm the formation of the polymer matrix, gel content analyses were carried out. The results showed high gel contents, up to 84 wt%, for most of the thermosets, except for MBDAC-BPPO 2 wt% P (Table 1). Indeed, the lowest gel contents were obtained for MBDAC-BPPO 1 wt% and 2 wt% P. The steric hindrance induced by the large BPPO group may reduce the reactivity and thus may lower the gel content.

DSC analyses were carried out to highlight the glass transition temperatures (T_g_) of the thermosets (Table 1). The reference containing only MBDAC exhibited a glass transition temperature at around 2 °C. This value is the lowest T_g_ compared to the other formulations. Thus, phosphorus moiety seems to favor higher T_g_. For MBDAC-DOPO, MBDAC-BPPO and MBDAC-DPP (Figures S29, S30 and S32) it is explained by the aromatic ring contained in the FR molecule. The OH group of the opened epoxy could also explain the slight increase [47,48]. Indeed, OH groups promote H-bonding between polymer chains which can explain the T_g_ increase. The increase in phosphorus amount showed various effects on T_g_. For example, with the MBDAC-DOPO, MBDAC-BPPO and MBDAC-DPP the T_g_ is lowered with 2 w% P. This can be due to a lower crosslinking density. However, using DEP, the T_g_ was improved. Interestingly the DPP exhibits two T_g_ meaning that the thermoset is heterogeneous. The second T_g_ inflexion is higher with 2 w% P meaning that it corresponds to the DPP FR.

The swelling rates were calculated and the values are presented in Table 1. The reference had a swelling rate of 190%, which was the lowest value obtained. Indeed, the MBDAC sample had the highest concentration in crosslinking nodes since all the cyclic carbonates were available to react. Thus, the solvent could not penetrate as much as with a lower concentration of crosslinking nodes. This trend is observed with the other thermosets. In fact, when DOPO, BPPO, DPP or DEP were grafted onto the MBDA, one epoxy is converted. This enables us to get three cyclic carbonates available to react instead of 4. Thus, the resulting thermosets had a lower density of crosslinking nodes. That is why when 1 w% P is used, the swelling rate is higher than that of the reference. Hence, 2 w% P led to a higher swelling rate than 1% P for all the samples. The swelling rate for the DOPO thermosets is significantly higher than for the other FR-thermosets.

2. Thermal and flame-retardancy analyses

Thermal and flame-retardancy properties were investigated in this part to highlight the influence of phosphorus structures on the material formulations and their modes of action. Indeed, phosphinate-containing molecule (DOPO) was compared to phosphonate-containing molecules (BPPO, DEP and DPP). Moreover, different phosphonate compounds were used in order to determine the influence of the structure on the thermal and flame-retardant properties.

- Thermogravimetric analyses

**Figure 2:**
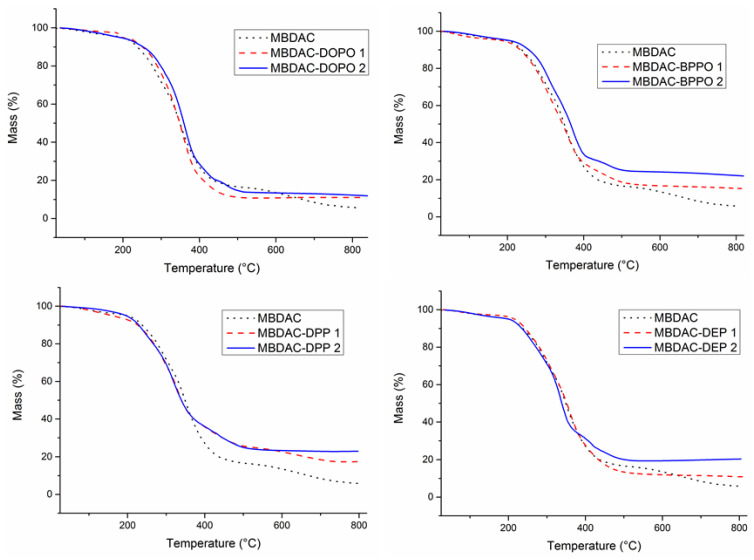
TGA thermograms of PHU materials.

The thermogravimetric analyses (TGA) under nitrogen flow, allowed us to determine the decomposition behavior of the different materials. Figure 2 shows the thermograms of the PHU materials with four different phosphorus-containing molecules (DOPO, BPPO, DEP, DPP) at various ratios of phosphorus (1 wt% and 2 wt%) compared to the free-phosphorus reference. Thermal parameters, such as the decomposition temperature at 5% weight loss (T_d_,_5wt%_), temperature at 50% weight loss (T_d,50wt%_) and the residue yield at 750 °C, are summarized in Table 2.

The T_d,5wt%_ values are reached around the same temperature, i.e., 200 °C for all the materials. Considering the temperature at 50 % weight loss (T_d,50wt%_), all the materials exhibited equivalent thermal stability with temperatures ranging between 338 °C and 366 °C. For all the samples, the curves exhibited two main decomposition steps. The first one appeared between 200 and 400 °C and could be attributed to the decomposition of the polymers. For the MBDAC polymer, the second step was observed between 500 and 750 °C; whereas, it was shifted to lower temperatures (400–500 °C) for the polymers containing phosphorus-containing functions. Moreover, as presented in Figure 2, the second step is more significant for the phosphonate than for the phosphinate compounds and it appeared at lower temperatures for phosphonate compounds. Indeed, the second step occurred between 370 and 400 °C for the PHU containing 2 wt% P phosphonate; whereas, it appeared around 440 °C for the polymer containing 2 wt% P phosphinate. The degradation of PHU containing 2 wt% P DPP seems more complex with a third decomposition step, occurring after 600 °C. Hence, the higher the phosphorus concentration, the slightly lower the temperature of the second step decomposition. The residue yield is improved with the introduction of phosphorus compounds. Moreover, it depends on both the phosphorus oxidation state and the structure of the compounds. Indeed, phosphonate compounds exhibited more residue content than phosphinate compounds. MBDAC-DOPO 2 wt% P exhibited a char yield of 12%, whereas MBDAC-BPPO, MBDAC-DPP and MBDAC-DEP displayed char contents of 22, 23 and 20%, respectively. Moreover, MBDAC-DPP and MBDAC-BPPO materials were composed of two more aromatic rings than MBDAC-DEP, which could explain a slightly higher residue content. These results were consistent with the mode of action of phosphonate compounds that act mainly in a condensed phase [49,50,51]. Indeed, phosphonate compounds may decompose earlier and participate to the formation of a char layer through cyclization, cross-linking and aromatization by dehydration of the polymeric structure [52,53]. Then, this carbon layer could insulate the polymer and prevent heat and gas transfer between gas and condensed phases [54,55]. Thereby, the carbon layer could protect the remaining polymer from further degradation.

During decomposition, the phosphorus-containing compounds may generate free radicals such as PO^●^, HPO^●^, PO2^●^. These radicals can quench the active radicals generated by the flame (H^●^ and OH^●^) and then, reduce the combustion in the gaseous phase. This mode of action was formerly observed for phosphinate compounds such as DOPO, in the literature [49,56]. Nevertheless, such a mode of action cannot be evidenced using TGA.

- Pyrolysis combustion flow calorimeter analysis

**Figure 3:**
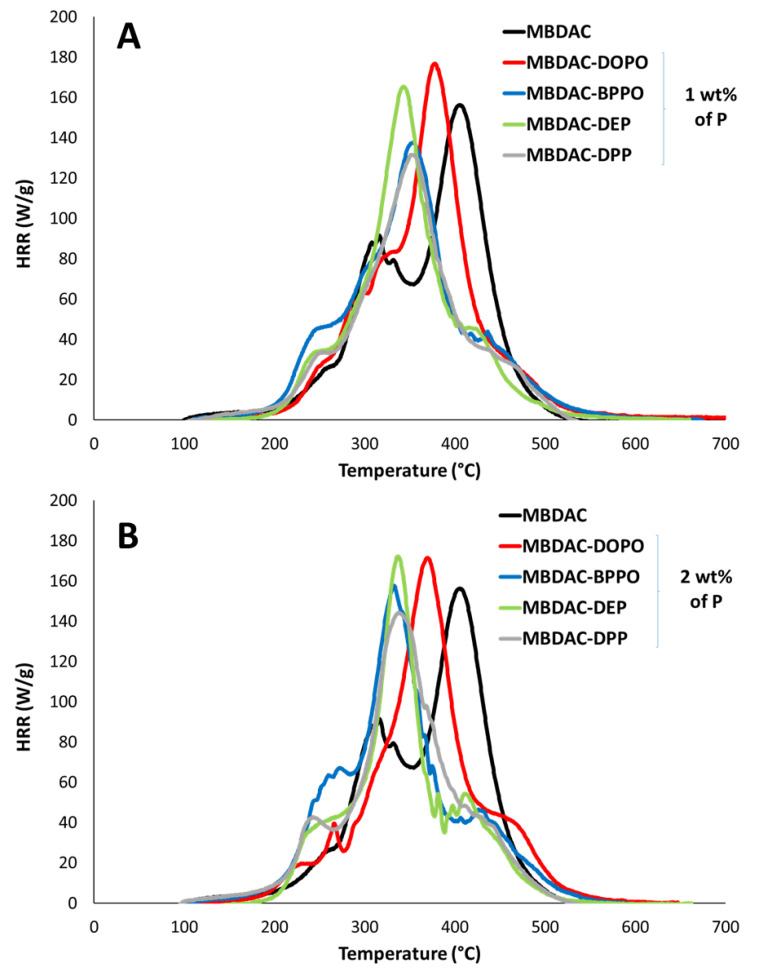
HRR curves for all PHUs in PCFC (**A**) with 1 wt% of phosphorus, (**B**) with 2 wt% of phosphorus.

The flammability of PHUs was investigated using PCFC. HRR curves are shown in Figure 3 and the main data are listed in Table 3.

All curves show that HRR starts to increase in the same range of temperatures (after 200 °C) in good agreement with T_d,5wt%_ measured in TGA. The decomposition occurs in several steps for all the PHUs. The first shoulder at a low temperature is more significant for BPPO-based PHUs. This may be ascribed to the lower crosslinking rate of these resins and then a higher amount of unreacted monomers could be released.

The main peak is located at a higher temperature, for reference (403 °C), than for phosphorus-based PHUs. Moreover, the temperature at pHRR is also slightly higher for DOPO-based PHUs than for phosphonate-based PHUs (>360 °C versus 330–350 °C) which is also expected. The intensity is in the range 130–170 W.g^−1^ for all resins, including the reference one. It may be slightly lower for the DPP compound.

Residue content is slightly enhanced for phosphorus-based PHUs, and slightly higher for phosphonate compounds than for phosphinate ones. Nevertheless, these residues are still lower than those noted from TGA analyses. The reason is unclear and may be due to the dependence of decomposition on the heating rate. Nevertheless, an intumescent char is sometimes observed, especially for DEP-based PHUs.

The heat of complete combustion is also in the same range, i.e., around 20 (±2) kJ.g^−1^. It is noteworthy that flame inhibition (which is the main mode of action of DOPO, for example) cannot be observed in standard conditions in PCFC, because the combustion is forced to be complete.

While the heat of combustion is rather constant and residue does not increase strongly, THR is also in the same range (16–19 kJ.g^−1^) for all PHUs. DEP seems to provide the lowest values due to the combination of a higher residue content and lower heat of combustion.

- Cone calorimeter test

**Figure 4:**
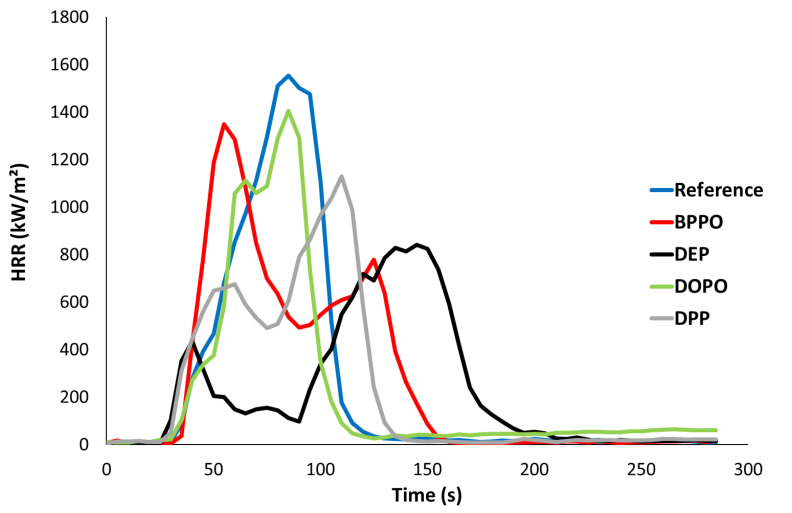
HRR curves in cone calorimeter for reference and PHUs containing 1wt% of phosphorus.

**Figure 5:**
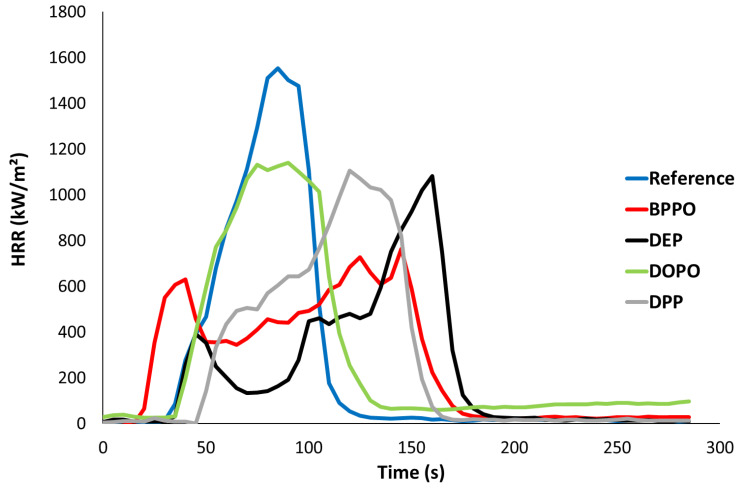
HRR curves in cone calorimeter for reference and PHUs containing 2 wt% of phosphorus.

Fire behavior at bench-scale was studied using q cone calorimeter (external heat flux 35 kW·m^−2^). Figure 4 and Figure 5 exhibit the HRR curves for resins containing 1 and 2 wt% of phosphorus, respectively. The main data are listed in Table 4 and Table 5. While residue contents are similar in PCFC and cone calorimeter, combustion efficiency X can be properly calculated as the ratio between effective heat of combustion in cone calorimeter (EHC) and heat of complete combustion in PCFC.

Reference ignites at 36 s and vigorous bubbling is observed at the sample surface. The heat release rate increases continuously up to a very high value (1644 kW·m^−2^) at 90 s. Then, HRR decreases quickly due to fuel depletion up to flame out. The residue fraction is negligible. This behavior is typical of a thermally thin behavior due to the small dimensions of the sample as well as the lack of a protective char layer formed during burning. Moreover, combustion is close to being complete (X~1).

DOPO-based PHUs exhibit similar behavior (vigorous bubbling and only one pHRR at around 90 s) even if the pHRR is slightly lower (1100–1300 kW·m^−2^). Flashes are observed before ignition, which occurs at the same time as the reference. Residue content is enhanced (around 10%) but it is not intumescent and charring seems to occur late. It is well known that DOPO is a poor char promoter and is especially efficient in fire tests as LOI or UL94. In these tests, ignition source is removed after few seconds and flame poisoning (the main mode of action of DOPO) may lead to fast flame extinction. On the contrary, under forced burning as in cone calorimeter, DOPO is quite often less efficient. Nevertheless, it should be noted that combustion efficiency is close to 1 even for DOPO-based PHUs. In other words, even flame poisoning does not occur for these DOPO-based PHUs.

Phosphonate-based PHUs exhibit a different behavior with two pHRRs. The first one is observed early, at 40–70 s, while the second one occurs at 120–165 s. Nevertheless, significant differences can be observed between these materials.

TTI is reduced for BPPO-based PHU (with 2 wt% of phosphorus). Vigorous bubbling is still observed for both materials (1 and 2 wt% P) and it explains why the first pHRR is intense. However, a char is formed with limited intumescence and reduces to some extent the HRR. Nevertheless, gases trapped under char are managed to be released leading to enhanced burning, corresponding to the pHRR2. As for DOPO compounds, residue content is close to 10% and combustion is complete.

On the contrary, DEP-based PHUs (and DPP-based PHUs to a lesser extent) show very different behavior. Ignition is reduced for 1 wt% P compounds (in comparison to the reference) but increases for 2 wt% P. No bubbling is observed and char is formed quickly after ignition leading to a low pHRR1. After pHRR1, the intumescent char efficiently traps the gases and the flame is strongly reduced and even vanishes in one case (one test for DEP-based PHU with 1 wt% P—see video in Supplementary Materials). Nevertheless, after several dozen seconds, gases are released at the bottom of the char and burning resumes forcefully leading to the pHRR2. Intumescence is especially significant for DEP-based PHUs and occurs in two steps corresponding roughly to both pHRRs. Residue fraction remains moderate (slightly higher with DEP compounds) and combustion is close to being complete in all cases (X > 0.9). It is noteworthy that the residue content is similar for all phosphorus-based PHUs. In other words, the cohesion of the char layer (for DEP, char’s interior is finely porous), as well as the time of its formation (early for DEP compounds, later for DOPO or BPPO ones), explains why DEP allows reducing more significantly HRR than other phosphorus compounds.

As for PCFC, DEP-based PHUs exhibit a slightly lower heat of combustion. Together with a higher residue fraction, they lead to a lower THR (around 15.5 kJ·g^−1^ versus 16.6–20 kJ·g^−1^ for other resins).

Finally, all phosphorus-based PHUs show enhanced smoke production. Total Smoke Production (TSR) increases from 820 m^2^·m^−2^ for the reference to more than 2000 m^2^·m^−2^ for DOPO-based PHUs. Smoke production basically depends on the material composition as well as on the burning rate. TSR is the highest for DOPO and BPPO, and lower for DEP and DPP, i.e., for the PHUs showing the lowest HRR. On another hand, TSR increases when phosphorus content increases from 1 to 2 wt% P (except for DEP).

**Figure 6:**
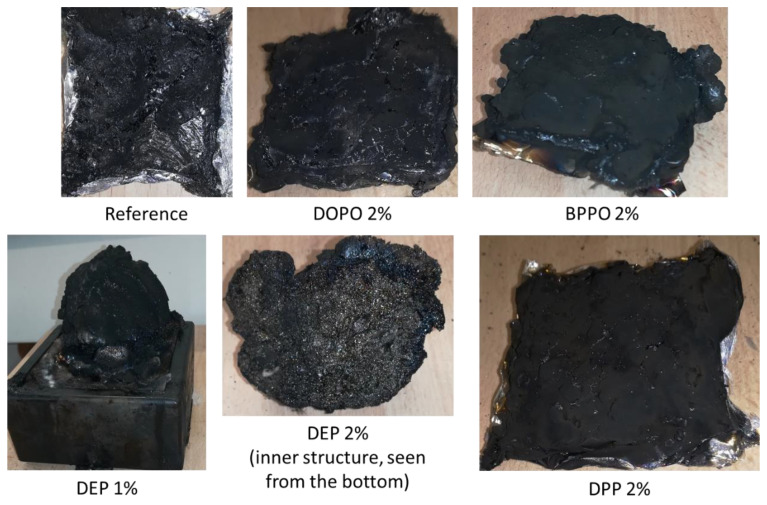
Pictures from some chars obtained after cone calorimeter tests.

Pictures of some chars from cone calorimeter tests are shown in Figure 6. The reference does not lead to any cohesive char covering the whole sample surface. Char from DOPO-based PHUs is more consistent but many cracks can be observed preventing its barrier effect. Chars from BPPO and DPP-based PHUs are only slightly expanded but rather cohesive. Nevertheless, chars from DEP-based PHUs are strongly intumescent allowing efficient protection of the underlying material from the heat flux. While the volume is much larger but the residue content is only slightly higher, such chars are much more porous. The inner structure is made of many small pores which must be beneficial for effective insulating protection.

## 3. Materials and Methods

### 3.1. Materials

9,10-dihydro-9-oxa-10-phosphaphenanthrene-10-oxide (DOPO) and 3,5-bis(trifluoromethyl)phenyl isothiocyanate (purity 98%) were acquired from TCI. Poly(propylene glycol) diglycidyl ether PPO GE (Mn = 640 g·mol^−1^), poly(methylhydrosiloxane) (PMHS, Mn = 2080 g·mol^−1^)[46], 4,4′-methylenebis(N,N-diglycidylaniline) (MBDA), tetrabutylammonium bromide (TBAB) (purity 99%), 2,2′-biphenol, phosphorus trichloride (PCl_3_), diphenyl phosphite (DPP), diethyl phosphite (DEP), anhydrous tetrahydrofuran, lithium bis(trimethylsilyl)amide solution (LiHMDS 1M in THF), boron trifluoride diethyl etherate (BF_3_.Et_2_O) and cyclohexylamine, were purchased from Sigma-Aldrich (Darmstadt, Germany). Jeffamine® EDR-148 (2,2′-(ethylenedioxy)bis(ethylamine)) was kindly supplied by Huntsman. CO_2_ was acquired from Air Liquide. Sodium hydroxide (NaOH), ethyl acetate (AcOEt), dioxane, tetrahydrofuran (THF) and dichloromethane (DCM) were purchased from VWR International S.A.S (Fontenay-sous-Bois, France). The NMR solvents used are CDCl_3_ and DMSO-d6 from Eurisotop.

### 3.2. Methods

#### 3.2.1. Nuclear Magnetic Resonance

NMR samples were prepared with CDCl_3_ or DMSO-d6 as solvents and the analyses were performed using a Bruker Avance 400 MHz spectrometer (Bruker, Billerica, MA, USA) at 25 °C. The structures of monomers were determined by hydrogen nuclear magnetic resonance (^1^H NMR), and phosphorus nuclear magnetic resonance (^31^P NMR). External references were trimethylsilane (TMS) for ^1^H and phosphoric acid (H_3_PO_4_) for ^31^P NMR. Shifts were given in ppm.

#### 3.2.2. Titration of the Epoxy Equivalent Weight by ^1^H NMR Spectroscopy

The Epoxy Equivalent Weight (EEW) is the amount of epoxy monomer needed for one equivalent of reactive epoxy function. It was determined by ^1^H NMR using an internal standard (benzophenone). Known weights of the reactant and benzophenone were poured into a NMR tube and 550 μL of CDCl_3_ were added. The EEW was determined using Equation (1) by comparing the integration value of the signals assigned to the benzophenone protons (7.5–7.8 ppm) with the integration of the signals arising from epoxy protons (2.57 ppm).

(1) $$\mathrm{EEW}=\frac{\int^{\;}\mathrm{PhCOPh}\times {\;H}_{\mathrm{epoxy}}}{\int^{\;}\mathrm{epoxy}\times {\;H}_{\mathrm{PhCOPh}}}\times \frac{m_{\mathrm{epoxy}}}{m_{\mathrm{PhCOPh}}}\times M_{\mathrm{PhCOPh}}$$

∫_PhCOPh_: integration of the signal from benzophenone protons; ∫_epoxy_: integration of the signals from protons in the α epoxy function; H_epoxy_: number of protons in the α of the epoxy function; H_PhCOPh_: number of benzophenone protons; m_epoxy_: epoxy mass; m_PhCOPh_: benzophenone mass; M_PhCOPh_: benzophenone molecular weight.

#### 3.2.3. Titration of the Amine Equivalent Weight of Jeffamine EDR-148 by ^1^H NMR

The Amine Equivalent Weight (AEW) is the Jeffamine amount needed for one equivalent of reactive amine function. It was determined by ^1^H NMR using an internal standard (benzophenone). Known weights of product and benzophenone were poured into a NMR tube and 550 μL of CDCl_3_ were added. The AEW was determined using Equation (2) by comparing the integration value of the signals assigned to the benzophenone protons (7.5–7.8 ppm) with the integration of the signals arising from amine moiety protons (2.67 ppm).

(2) $$\mathrm{AEW}=\frac{\int^{\;}\mathrm{PhCOPh}\times {\;H}_{\mathrm{amine}}}{\int^{\;}\mathrm{amine}\times {\;H}_{\mathrm{PhCOPh}}}\times \frac{m_{\mathrm{amine}}}{m_{\mathrm{PhCOPh}}}\times M_{\mathrm{PhCOPh}}$$

∫_PhCOPh_: integration of the signal from benzophenone protons; ∫_amine_: integration of the signals from protons in α of the amine function; H_amine_: number of protons in α of the amine function; H_PhCOPh_: number of benzophenone protons; m_amine_: amine mass; m_PhCOPh_: benzophenone mass; M_PhCOPh_: benzophenone molecular weight.

#### 3.2.4. Titration of the Carbonate Equivalent Weight by ^1^H NMR

The Carbonate Equivalent Weight (CEW) is the amount of carbonate monomer needed for one equivalent of reactive cyclic carbonate function. It was determined by ^1^H NMR using an internal standard (benzophenone). Known masses of product and benzophenone were poured into a NMR tube and 550 μL of CDCl_3_ were added. The CEW was determined using Equation (3) by comparing the integration value of the signals assigned to the benzophenone protons (7.5–7.8 ppm) with the integration of the signals arising from cyclic carbonate (4.89 ppm for MBDAC and 4.5 ppm for PPO DC).

(3) $$\mathrm{CEW}=\frac{\int^{\;}\mathrm{PhCOPh}\times {\;H}_{\mathrm{carbonate}}}{\int^{\;}\mathrm{carbonate}\times {\;H}_{\mathrm{PhCOPh}}}\times \frac{m_{\mathrm{carbonate}}}{m_{\mathrm{PhCOPh}}}\times M_{\mathrm{PhCOPh}}$$

∫_PhCOPh_: integration of the signal from benzophenone protons; ∫_carbonate_: integration of the signals from protons in α of the carbonate function; H_carbonate_: number of protons in α of the carbonate function; H_PhCOPh_: number of benzophenone protons; m_carbonate_: product mass; m_PhCOPh_: benzophenone mass; M_PhCOPh_: benzophenone molecular weight.

#### 3.2.5. Fourier Transform Infrared Spectroscopy

The Fourier transform infrared spectroscopy (FTIR) spectra were acquired on a Thermo Scientific Nicolet iS50 FT-IR equipped with an attenuated total reflectance cell (ATR). The data were analyzed using the software OMNIC Series 8.2 from Thermo Scientific.

#### 3.2.6. Differential Scanning Calorimetry

Differential Scanning Calorimetry (DSC) analyses were carried out using a NETZSCH DSC200F3 calorimeter, which was calibrated using Indium, n-octadecane n-octane, adamantane, biphenyl, Tin, Bismuth and Zinc standards. Nitrogen was used as a purge gas. Approximately 10 mg of sample were placed in a perforated aluminum pan and the calorific capacities were recorded between −150 °C and 120 °C at 20 °C·min^−1^ to observe the glass transition temperature (T_g_). The T_g_ values were measured on the second heating ramp to erase the thermal history of the polymer in the inflexion jump of the heat capacity. All the reported temperatures are average values on three different experiments.

#### 3.2.7. Gel Content and Swelling Rate

Three samples from the same material, of ca. 20 mg each, were separately immersed in THF for 24 h. The three samples were then wiped off THF and weighed to determine the swelling rate (SWI). The SWI was calculated using Equation (4) where m_1_ is the initial mass of the sample and m_2_ is the mass of the wet samples. Then, the samples were dried in a ventilated oven at 70 °C for 24 h. The gel content (GC) was calculated using Equation (5), where m_3_ is the mass of the dried material and m_1_ is the initial mass. Reported gel content is the average values of three samples.

(4) $$\mathrm{SWI}=\frac{m_{2}}{m_{1}}\times 100$$

(5) $$\mathrm{GC}=\frac{m_{3}}{m_{1}}\times 100$$

#### 3.2.8. Thermogravimetric Analysis

Thermogravimetric analyses (TGA) of the cured materials were carried out to determine the thermal stability and were performed on a Netzsch TG 209F1 apparatus (Netzsch Holding, Selb, Germany) under 40 mL.min^−1^ nitrogen flow. The protective gas used was nitrogen with a 20 mL.min^−1^ flow. Approximately 10–12 mg of sample were placed in an alumina crucible and heated from room temperature to 800 °C with a 20 °C.min^−1^ heating rate.

#### 3.2.9. Pyrolysis Combustion Flow Calorimeter Analysis

The fire behavior of resins was analyzed using a pyrolysis combustion flow calorimeter (PCFC - Fire Testing technology, East Grinstead, UK). About 3–4 mg were placed in the pyrolyzer, undergoing an increase in temperature from 20 °C to 750 °C at a rate of 1 °C·s^−1^ under a nitrogen flow. Pyrolytic gases were sent to a combustor heated at 900 °C under airflow (N_2_/O_2_ = 80/20). At this temperature and with 20% oxygen, combustion was considered to be completed. HRR was determined according to oxygen depletion (Huggett’s relation) as the cone calorimeter test. PCFC analyses correspond to anaerobic pyrolysis followed by the high-temperature oxidation of decomposition products (complete combustion) [57]. All samples were tested in duplicate.

#### 3.2.10. Cone Calorimeter Test

Flammability was studied using a cone calorimeter (Fire Testing technology, East Grinstead, United Kingdom) in order to investigate the fire behavior of materials. The PHU sample dimensions were 50 × 50 mm^2^. All the samples exhibited a weight of 10 (±0.4) g. The samples were placed at 2.5 cm below the conic heater and exposed to a 35 kW·m^−2^ heat flux in well-ventilated conditions (air rate 24 L·s^−1^) in the presence of a spark igniter to force the ignition. Heat-release rate (HRR) was determined by oxygen depletion according to the Huggett principle (1 kg of consumed oxygen corresponds to 13.1 MJ of heat released) [58]. While the exposed sample surface is lower than that of the sample holder (100 mm × 100 mm), the samples were carefully coated with aluminum foil and placed in rockwool to avoid that heat flux was absorbed by the sample edges.

### 3.3. Syntheses

#### 3.3.1. Synthesis of 1-(3,5-bis(trifluoromethyl)phenyl)-3-cyclohexylthiourea (Thiourea) [49]

**Figure 7:**
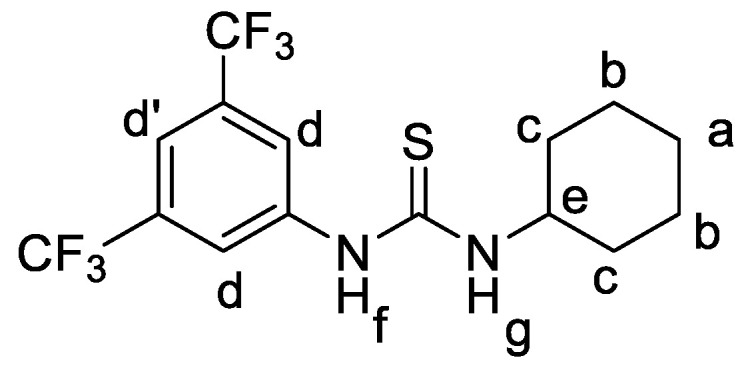
Thiourea structure.

Thiourea, represented in Figure 7, was synthesized according to the literature procedure at room temperature, and cyclohexylamine (14.63 g, 147.50 mmol) was added dropwise to a stirred solution of 3,5-bis(trifluoromethyl)phenyl isothiocyanate (40.00 g, 147.50 mmol) in THF (70 mL) [59]. After stirring for 4 h at room temperature, the solvent was evaporated. The white residue was recrystallized from chloroform to give thiourea as a white powder with a 85 % yield.

^1^H NMR (400 MHz, DMSO-d6, ppm): δ = 1.44 (m, 4H, H_c_), 1.5-1.99 (m, 4H, H_b_), 2.66 (m, 3H, H_e_ and H_a_), 7.75 (s, 4H, H_d_.), 8.18 (s, 1H, NH_g_), 8.39 (s, 2H, H_d’_), 9.89 (s, 1H, NH_f_). (Figure S1).

#### 3.3.2. Synthesis of 4,4′-(3,6,9,12,15,18,21-heptamethyl-2,5,8,11,14,17,20,23-octaoxatetracosane-1,24-diyl)bis(1,3-dioxolan-2-one) (PPO DC) [59]

**Figure 8:**
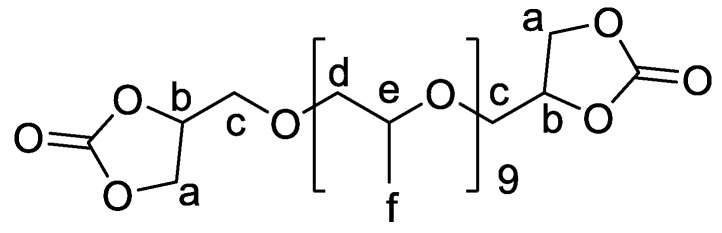
PPO DC structure.

The PPO DC was synthetized according to the method used by Cornille et al. [59]. Poly(propylene glycol) diglycidyl ether (PPO GE 640; 300 g, 0.468 mol) and tetra-butylammonium bromide (TBAB; 4.53 g, 0.029 mmol) were solubilized in 200 mL of AcOEt and added to a 600 mL sealed reactor. The reaction was carried out at 80 °C under 20 bars of CO_2_ for 120 h. The crude mixture was then washed with water and brine to remove TBAB. The organic layer was then dried with magnesium sulfate and under vacuum. The pure product, presented in Figure 8, was obtained as a yellowish viscous liquid with a 93% yield.

^1^H NMR (400 MHz, CDCl_3_, ppm): δ = 1.15 (d, 21H, CH_3_, H_f_), 3.33–3.95 (m, 25H, H_c_, H_d_ and H_e_), 4.5 (m, 4H, CH_2_, Ha), 4.8 (t, 2H, CH, H_b_). (Figure S2).

#### 3.3.3. Synthesis of 4,4′,4″,4‴-(((methylenebis(4,1-phenylene))bis(azanetriyl))tetrakis (methylene))tetrakis(1,3-dioxolan-2-one) (MBDAC) [45]

**Figure 9:**
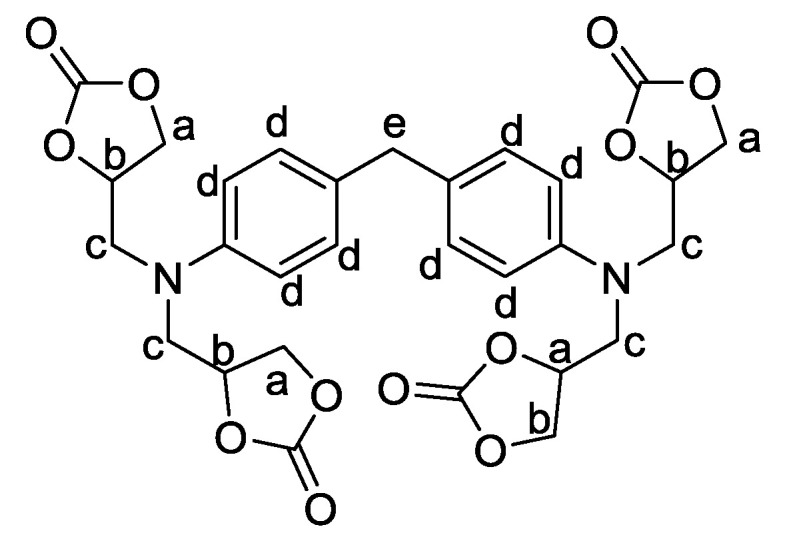
MBDAC structure.

The MBDAC-DOPO was synthetized according to the method used by Coste et al. [45]. 4,4′-Methylenebis(N,N-diglycidylaniline) (Figure S3) (100 g, 0.236 mol) and tetrabutylammonium bromide (2.29 g, 0.007 mmol) were solubilized in 150 mL of dichloromethane (DCM) and added to a 300 mL sealed reactor. The reaction was carried out at 80 °C under 20 bars of CO_2_ for 120 h. The crude mixture was then washed with water and brine to remove TBAB. The organic layer was then dried with magnesium sulfate and under vacuum. The pure product (Figure 9) was obtained as a yellowish viscous liquid with a 81% yield.

^1^H NMR (400 MHz, DMSO-d6, ppm): δ = 3.92 (m, 10H, H_c_ and H_e_), 4.27 (m, 4H, H_a_), 4.51 (m, 4H, H_a_), 4.89 (m, 4H, H_b_), 6.72 (m, 4H, H_d_), 7.10 (m, 4H, H_d_). (Figure S4).

#### 3.3.4. Synthesis of 6-(3-((4-(4-(bis(oxiran-2-ylmethyl)amino)benzyl)phenyl)(oxiran-2-ylmethyl) amino)-2-hydroxypropyl)dibenzo[c,e][1,2]oxaphosphinine 6-oxide (MBDA-DOPO) [45]

**Figure 10:**
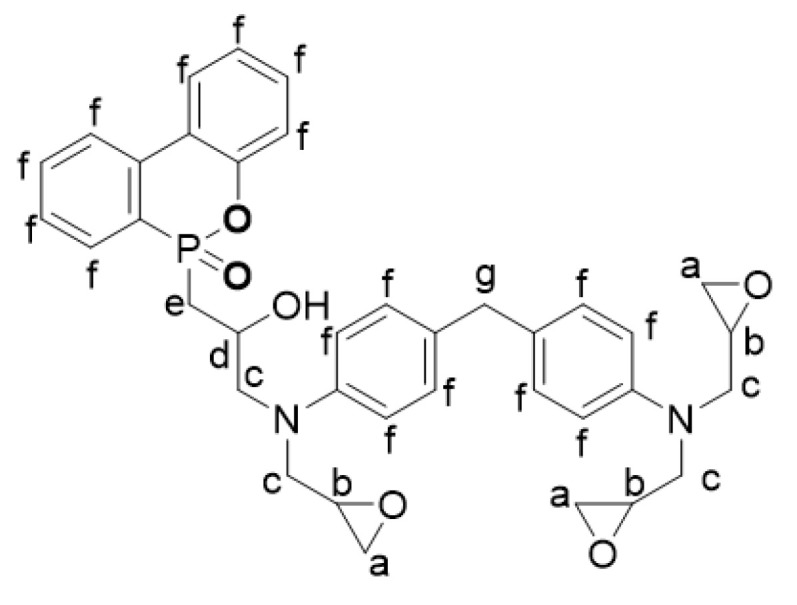
MBDA-DOPO structure.

The MBDA-DOPO was synthetized according to the method used by Coste et al. [45]. In a 500 mL three-necked round-bottom flask equipped with a condenser and a mechanical stirrer, 100 g (0.237 mol, 1 eq.) of MBDAC and 51.12 g (0.237 mol, 1 eq.) of DOPO were introduced and purged with N_2_ during 30 min. Then, the reaction mixture was allowed to heat at 155 °C for 6h. In the end, the reaction was frozen and ground to obtain a pale orange powder of MBDA-DOPO (Figure 10) in 97% yield. The phosphorus ratio was 5 wt% in this flame-retardant product and the EEW was 3.

^1^H NMR (400 MHz, DMSO-d6, ppm): δ = 2.27 (m, 2H, H_e_), 2.61 (m, 3H, H_a_), 2.79 (m, 3H, H_b_), 3.17 (m, 3H, H_a_), 3.45 (m, 4H, H_c_), 3.70–3.80 (dt, 5H, H_c_, H_d_), 3.82 (s, 2H, H_g_), 6.68–8.20 (m, 16H, H_f_). (Figure S7).

^31^P NMR (161.6 MHz, DMSO-d6, ppm): δ = 36.04 ppm. (Figure S8).

HRMS (ESI+): calc. for [M + H^+^] 639.25 g·mol^−1^, found 639.26 g·mol^−1^.

#### 3.3.5. Synthesis of 4,4′-(((4-(4-((2-hydroxy-3-(6-oxidodibenzo[c,e][1,2]oxaphosphinin-6-yl)propyl) ((2-oxo-1,3-dioxolan-4-yl)methyl)amino)benzyl)phenyl)azanediyl)bis(methylene))bis(1,3-dioxolan-2-one) (MBDAC-DOPO) [45]

**Figure 11:**
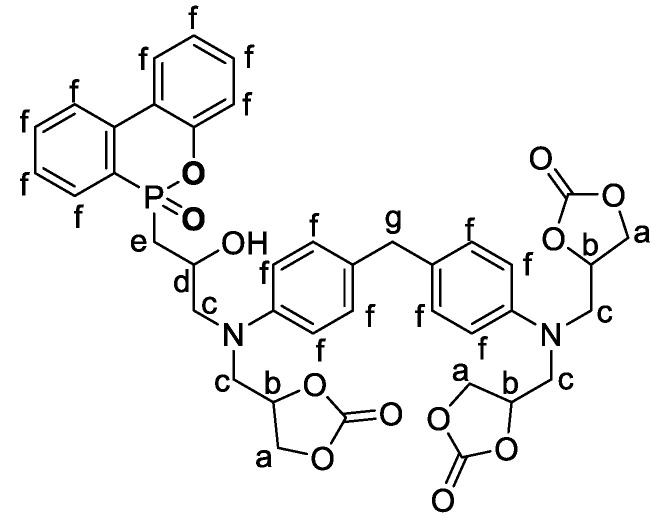
MBDAC-DOPO structure.

The MBDAC-DOPO was synthetized according to the method used by Coste et al. [45] MBDA-DOPO (200 g, 0.313 mol) and tetrabutylammonium bromide (3.03 g, 0.009 mol) were solubilized in 300 mL of dichloromethane (DCM) and added to a 300 mL sealed reactor. The reaction was carried out at 80 °C under 20 bars of CO_2_ for 120 h. The crude mixture was then washed with water and brine to remove TBAB. The organic layer was then dried with magnesium sulfate and under vacuum. The pure product, presented in Figure 11, was obtained as a yellowish viscous liquid with a 78% yield. The MBDAC-DOPO exhibited a phosphorus percentage of 4.0%.

^1^H NMR (400 MHz, DMSO-d6, ppm): δ = 3.50−3.95 (m, 10H, H_e_, H_C_), 4.17 (m, 3H, H_a_), 4.55 (m, 3H, CH_b_), 4.79 (m, 3H, H_a_), 6.55–8.30 (m, 15H, H_f_). (Figure S9).

^31^P NMR (161.6 MHz, DMSO-d6, ppm): δ = 36.04 ppm. (Figure S10).

HRMS (ESI+): calc. for [M + H^+^] 771.22 g.mol^−1^, found 771.23 g.mol^−1^.

Different hydroxyphosphonates were reacted with epoxy MBDA, following the synthesis procedure of Ghoteimi et al. [60].

#### 3.3.6. Synthesis of Diethyl (3-((4-(4-(bis(oxiran-2-ylmethyl)amino)benzyl)phenyl)(oxiran-2-ylmethyl)amin-o)-2-hydroxypropyl)phosphonate (MBDA-DEP)

**Figure 12:**
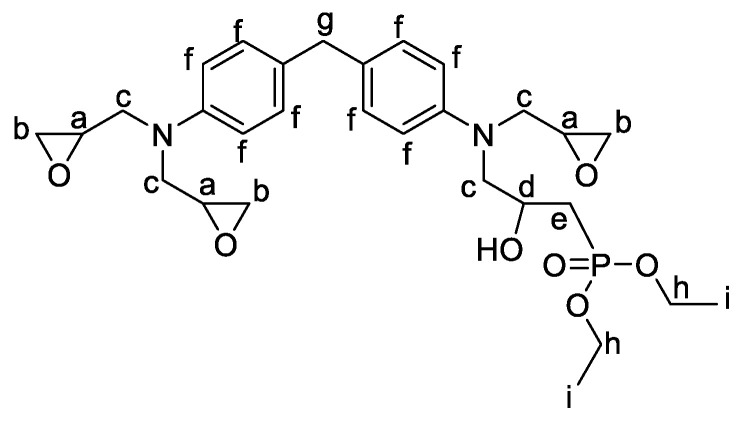
MBDA-DEP structure.

In a 250 mL three-necked round-bottom flask, diethyl phosphite (10 g, 0.072 mol) was introduced and then solubilized with 25 mL of anhydrous THF. The solution was frozen using liquid nitrogen and degassed under a vacuum before being backfilled with N_2_. Three cycles were carried out. The mixture was allowed to warm up to room temperature. Then, the reaction was cooled to −78 °C using liquid nitrogen and acetone. A solution of LiHMDS 1M in THF (72 mL, 0.072 mol) was added dropwise with a syringe pump. After the completed addition, a solution of the epoxide MBDA (30.42 g, 0.072 mol) in 50 mL of anhydrous THF was added, followed by the addition of BF_3_.Et_2_O (10.2 g, 0.072 mol). The reaction mixture was stirred at −78 °C for an additional 30 min, then allowed to warm up to room temperature and stirred overnight. The mixture was cooled to −15 °C and the reaction was quenched by adding a saturated solution of NH_4_Cl. The aqueous layer was extracted with DCM (3 × 150 mL). Then, the combined organic layers were dried over MgSO_4_, filtered and the solvent was removed under reduced pressure. The pure product, presented in Figure 12, was obtained as a pale pink powder with a 88% yield.

^1^H NMR (400 MHz, DMSO-d6, ppm): δ = 1.36 (m, 6H, H_i_), 1.8-2.1 (m,2H, H_e_), 2.45 (m, 3H, H_b_), 2.53 (m, 3H, H_a_), 2.68 (m, 3H, H_b_), 3.10-3.72 (m, 7H, H_c_, H_d_), 3.82-4.19 (m, 6H, H_g_, H_h_), 6.76-7.15 (m, 8H, H_ar_ (Figure S19).

^31^P NMR (161.6 MHz, DMSO- d6, ppm): δ = 29.6 ppm. (Figure S20).

HRMS (ESI+): calc. for [M + H^+^] 561.27 g·mol^−1^, found 561.27 g·mol^−1^.

#### 3.3.7. Synthesis of Diethyl (3-((4-(4-(bis((2-oxo-1,3-dioxolan-4-yl)methyl)amino)benzyl)phenyl)((2-oxo-1,3-dioxolan-4-yl)methyl)amino)-2-hydroxypropyl)phosphonate (MBDAC-DEP)

**Figure 13:**
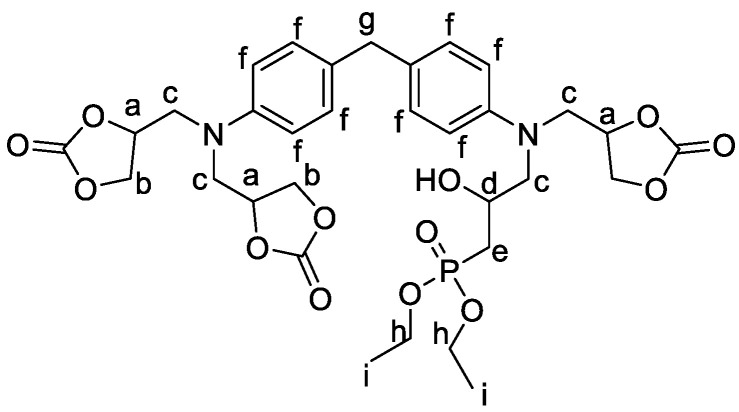
MBDAC-DEP structure.

MBDA-DEP (100 g, 0.18 mol) and tetrabutylammonium bromide (2.9 g, 0.009 mol) were solubilized in 300 mL of dichloromethane (DCM) and added to a 600 mL sealed reactor. The reaction was carried out at 80 °C under 20 bars of CO_2_ for 120 h. The crude mixture was then washed with water and brine to remove TBAB. The organic layer was then dried with magnesium sulfate and under vacuum. The pure product, presented in Figure 13, was obtained as a pale pink powder with a 76% yield. The MBDAC-DEP exhibited a phosphorus percentage of 4.5%.

^1^H NMR (400 MHz, DMSO-d6, ppm): δ = 1.25 (m, 6H, H_i_), 2.34 (m (small), 2H, H_e_), 3.4–3.82 (m, 5, H_c_, H_d_), 3.9–4.1 (m, 4H, H_a_, H_g_), 4.18 (m, 3H, H_b_), 4.58 (q, 4H, H_h_), 4.9 (m, 3H, H_a_), 6.7–7.1 (m, 8H, H_f_). (Figure S21).

^31^P NMR (161.6 MHz, DMSO-d6, ppm): δ = 29.6 ppm. (Figure S22).

HRMS (ESI+): calc. for [M + H^+^] 693.23 g·mol^−1^, found 693.25 g·mol^−1^.

#### 3.3.8. Synthesis of Diphenyl (3-((4-(4-(bis(oxiran-2-ylmethyl)amino)benzyl)phenyl)(oxiran-2-ylmethyl)amino)-2-hydroxypropyl)phosphonate (MBDA-DPP)

**Figure 14:**
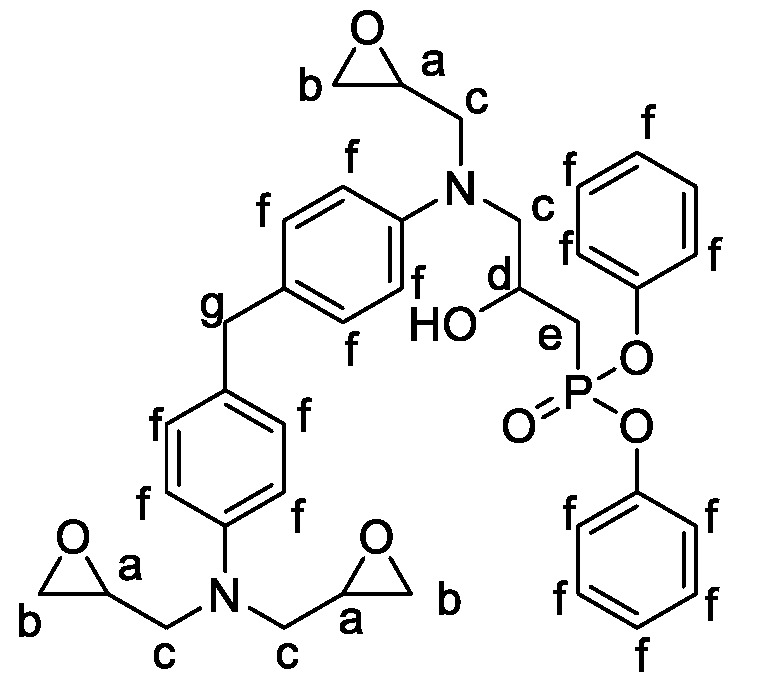
MBDA-DPP structure.

In a 250 mL three-necked round-bottom flask, 25 mL of anhydrous THF was introduced to solubilize diphenyl phosphite (20 g, 0.085 mol) previously introduced. The solution was frozen using liquid nitrogen and degassed under a vacuum before being backfilled with N_2_. Three cycles were carried out. The mixture was warmed up to room temperature, before the reaction was cooled to –78 °C using liquid nitrogen and acetone. A solution of LiHMDS 1M in THF (85 mL, 0.085 mol) was added dropwise with a syringe pump. After addition, a solution of MBDA (35.9 g, 0.085 mol) in 50 mL of anhydrous THF was added. Then, BF_3_.Et_2_O (12.1 g, 0.085 mol) was added. The reaction mixture was stirred at −78 °C for 30 min, then allowed to warm up to room temperature and stirred overnight at 20 °C. The mixture was cooled to −15 °C and the reaction was quenched by adding a saturated solution of NH_4_Cl. The aqueous layer was extracted with DCM (3 × 150 mL). Then, the combined organic layers were dried over MgSO_4_, filtered and the solvent was removed under reduced pressure. The pure product, presented in Figure 14, was obtained as a pale orange powder with a 82% yield.

^1^H NMR (400 MHz, DMSO-d6, ppm): δ = 1.75 (m, 2H, H_e_), 2.55 (m, 3H, H_a_), 2.72 (m, 3H, H_b_), 3.11 (m, 3H, H_a_), 3.15 (m, 4H, H_c_), 3.30–3.8 (m, 7H, H_c_, H_g_, H_d_), 6. 21–7.50 (m, 18H, H_f_). (Figure S25).

^31^P NMR (161.6 MHz, DMSO-d6, ppm): δ = 23.4 ppm. (Figure S26).

HRMS (ESI+): calc. for [M + H^+^] 657.27 g·mol^−1^, found 657.28 g·mol^−1^.

#### 3.3.9. Synthesis of Diphenyl (3-((4-(4-(bis((2-oxo-1,3-dioxolan-4-yl)methyl)amino)benzyl)phenyl)((2-oxo-1,3-dioxolan-4-yl)methyl)amino)-2-hydroxypropyl)phosphonate (MBDAC-DPP)

**Figure 15:**
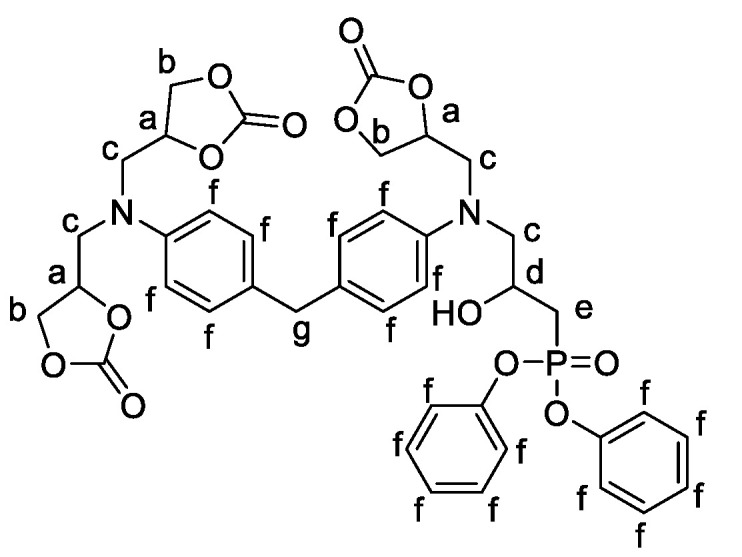
MBDAC-DPP structure.

MBDA-DPP (100 g, 0.15 mol) and tetrabutylammonium bromide (2.6 g, 0.008 mol) were solubilized in 300 mL of dichloromethane (DCM) and added to a 600 mL sealed reactor. The reaction was carried out at 80 °C under 20 bars of CO_2_ for 120 h. The crude mixture was then washed with water and brine to remove TBAB. The organic layer was then dried with magnesium sulfate and under vacuum. The pure product, presented in Figure 15, was obtained as a pale brown powder with a 72% yield. The MBDAC-DPP exhibited a phosphorus percentage of 4.0%.

^1^H NMR (400 MHz, DMSO-d6, ppm): δ = 1.6 (m, 2H, H_e_), 3.25–3.80 (m, 9H, H_c_, H_d_), 4.2 (m, 3H, H_b_), 4.6 (m, 3H, H_a_), 4.92 (m, 3H, H_b_), 6.57–7.47 (m, 18H, H_f_). (Figure S27).

^31^P NMR (161.6 MHz, DMSO-d6, ppm): δ = 23.4 ppm. (Figure S28).

HRMS (ESI+): calc. for [M + H+] 789.23 g·mol^−1^, found 789.26 g·mol^−1^.

#### 3.3.10. Synthesis of Dibenzo[d,f][1,3,2]dioxaphosphepine 6-oxide (BPPO) [61]

**Figure 16:**
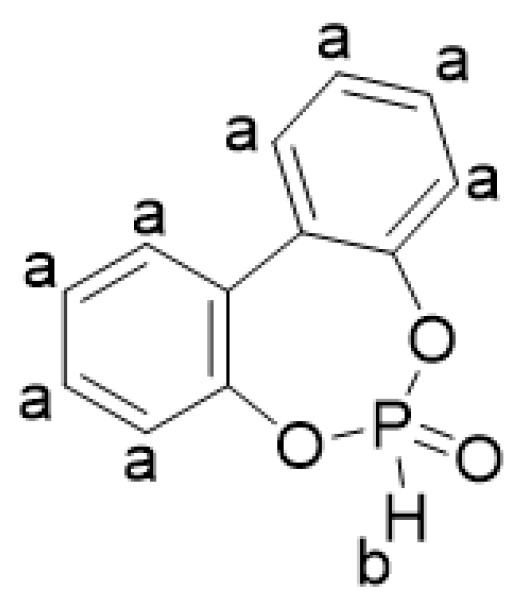
BPPO structure.

The BPPO was synthetized according to the method used by Lenz et al. [61]. In a 1000 mL three-necked round-bottom flask equipped with a condenser, a magnetic stirring bar, a dropping funnel and a nitrogen inlet, 200 g of 2,2′-biphenol (1.07 mol, 1 eq.) and 19.26 g of water (1.07 mol, 1 eq.) were dissolved in 500 mL of 1,4-dioxane and heated at 100 °C. A continuous flow of nitrogen was passed through the solution. Then, the dropping funnel was filled with 147.5 g of PCl_3_ (1.07 mol, 1 eq.). Within 4h, PCl_3_ was slowly added to the boiling reaction mixture. A trap containing a concentrated NaOH solution was added to absorb the generated HCl gas. After the total completion of the addition of PCl_3_, the mixture was heated to reflux for an additional hour. Then, the solvent was removed under reduced pressure and a pale brown powder was obtained. The product was washed three times with diethyl ether and dried under reduced pressure to give BPPO in 92% yield (Figure 16).

^1^H NMR (400 MHz, DMSO-d6, ppm): δ = 8.53 and 6.62 (s, J = 324.7 H_z_, 1H, H_b_), 7.79–7.39 (m, 8H, H_a_). (Figure S11).

^31^P NMR (161.6 MHz, DMSO-d6, ppm): δ = 15.9 ppm. (Figure S12).

#### 3.3.11. Synthesis of 6-(1-((4-(4-(bis(oxiran-2-ylmethyl)amino)benzyl)phenyl)(oxiran-2-ylmethyl)amino)-3-hydroxypropan-2-yl)dibenzo[d,f][1,3,2]dioxaphosphepine 6-oxide (MBDA-BPPO)

**Figure 17:**
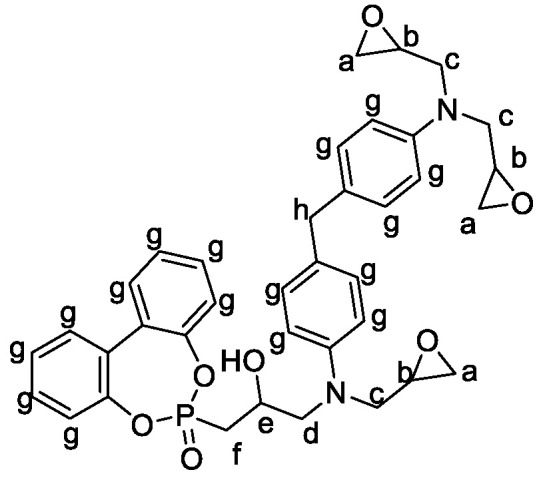
MBDA-BPPO structure.

In a 500 mL three-necked round-bottom flask, BPPO (20 g, 0.086 mol) was introduced and 50 mL of anhydrous THF was added to solubilize BPPO powder. The solution was frozen with liquid nitrogen and degassed under a vacuum before being backfilled with N_2_. Three cycles were carried out. The mixture was allowed to warm up to room temperature. Then, the reaction was cooled to –78 °C using liquid nitrogen and acetone. A solution of LiHMDS 1M in THF (86 mL, 0.086 mol) was added dropwise with a syringe pump. After completed addition, a solution of the epoxide MBDA (36.42 g, 0.086 mol) in 75 mL of anhydrous THF was added, followed by the addition of BF_3_.Et_2_O (12.2 g, 0.086 mol). The reaction mixture was stirred at −78 °C for 30 min, then warmed up to room temperature and stirred for 15 h. The mixture was cooled to −15 °C and the reaction was quenched by adding a saturated solution of NH_4_Cl. The aqueous layer was extracted with DCM (3 × 300 mL). Then, the combined organic layers were dried over MgSO_4_, filtered and the solvent was removed under reduced pressure. The pure product (Figure 17, was obtained as an orange–brown powder with a 85% yield.

^1^H NMR (400 MHz, DMSO-d6, ppm): δ = 1.15 (m, 2H, H_f_), 2.42 (m, 3H, H_a_), 2.52 (m, 3H, H_b_), 2.68 (m, 3H, H_a_), 3.2–3.6 (m, 8H, H_e_, H_c_, H_h_), 6.6–7.7 (m, 15H, H_g_). (Figure S13).

^31^P NMR (161.6 MHz, DMSO-d6, ppm): δ = 39.1 ppm. (Figure S14).

HRMS (ESI+): calc. for [M + H^+^] 655.25 g·mol^−1^, found 655.26 g·mol^−1^.

#### 3.3.12. Synthesis of 4,4′-(((4-(4-((3-hydroxy-2-(6-oxidodibenzo[d,f][1,3,2]dioxaphosphepin-6-yl)propyl)((2-oxo-1,3-dioxolan-4-yl)methyl)amino)benzyl)phenyl)azanediyl) bis(methylene))bis(1,3-dioxolan-2-one) (MBDAC-BPPO)

**Figure 18:**
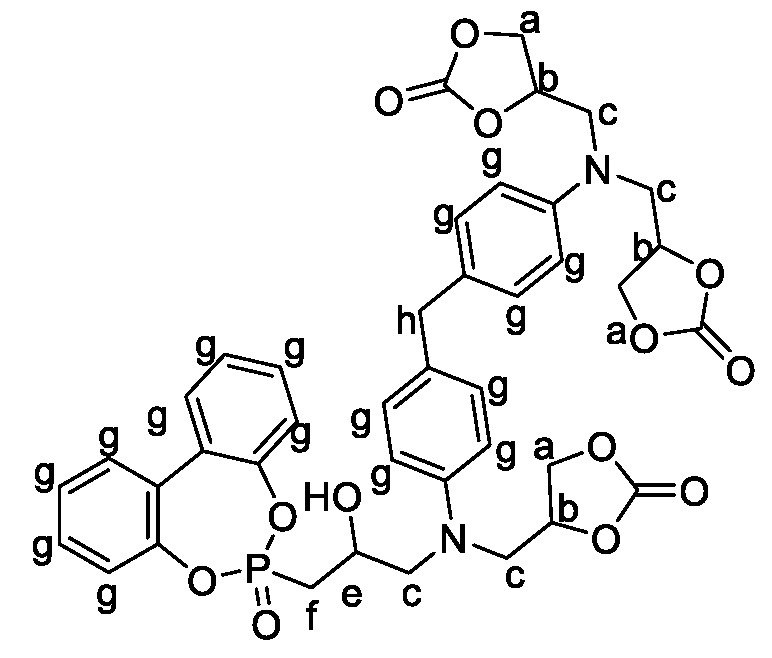
MBDAC-BPPO structure.

MBDA-BPPO (100 g, 0.153 mol) and tetrabutylammonium bromide (2.6 g, 0.008 mol) were solubilized in 300 mL of dichloromethane (DCM) and added to a 600 mL sealed reactor. The reaction was carried out at 80 °C under 20 bars of CO_2_ for 120 h. The crude mixture was then washed with water and brine to remove TBAB. The organic layer was then dried with magnesium sulfate and under vacuum. The pure product, presented in Figure 18, was obtained as a pale brown powder in 75% yield. The MBDAC-BPPO exhibited a phosphorus percentage of 4.0 wt%.

^1^H NMR (400 MHz, DMSO-d6, ppm): δ = 1.85 (m, 2H, H_f_), 3.17–4.2 (m, 20H H_a_, H_b_, H_c_, H_e_, H_h_), 6.55–7.75 (m, 16H, H_g_). (Figure S15).

^31^P NMR (161.6 MHz, DMSO-d6, ppm): δ = 40.02 ppm. (Figure S16).

HRMS (ESI+): calc. for [M + H^+^] 787.22 g·mol^−1^, found 787.23 g·mol^−1^.

### 3.4. Formulations of the PHU Materials

All the PHU materials were prepared with the same following procedure and exact masses were presented in supporting information (SI). The functionality of carbonate monomers used in the formulations of PHU materials is presented in Table 6. The desired amounts of phosphorus-containing compounds and 0.090 mol (1 eq.) of cyclic carbonates were introduced in vial with 5 wt% of the catalyst. The mixture was stirred with a SpeedMixer® for 2 min at 2500 rpm. Then, 0.090 mol (1 eq.) of the amine (EDR-148) was added and mixed for 30 s at 2500 rpm thanks to the SpeedMixer®. A total of 10 g of the mixture were poured into a 50 × 50mm silicon mold and heated at 80 °C for 24 h and then 150 °C for 2 additional hours. All formulations were composed of a tetrafunctional carbonate (MBDAC) and/or MBDAC modified with phosphorus group (PHUs with 1 and 2 wt% P), PPO DC, EDR 148, and catalyst (0.03 eq.). In order to run fire tests, each formulation was prepared four times to have three materials for cone calorimeter analysis and one for thermal and mechanical analysis.

## 4. Conclusions

In this study, different phosphorylated reagents (DEP, DOPO, DPP, BPPO) were grafted onto a cyclic carbonate (MBDAC) by an epoxy opening to create the original FR. Then, the multifunctional phosphorus-modified cyclic carbonate associated with the phosphorus-free cyclic carbonate, reacted at 90 °C with the Jeffamine EDR 148 to form the polymer network. The high gel contents obtained confirm the grafting of the FR on the polymer backbone and the polymer network formation. The thermosets were characterized by FTIR to confirm the conversion of the cyclic carbonate. DSC analyses were carried out to determine the glass transition of the different thermosets and the influence of the FR on the physical properties. Finally, the thermal stability and the fire behavior of the materials were analyzed by TGA, cone calorimeter and PCFC. First, the influence of the addition of FR was clearly observed by a decrease in the pHRR from 1644 kW·m^−^² to 1362 kW·m^−^² with MBDAC-DOPO 1 wt% P and even 427 kW·m^−^² with MBDAC DEP 1 wt% P. The phosphonate FR provided better results than phosphinate FR. Especially, MBDAC-DEP leads to the early formation of an intumescent char allowing a significant decrease in HRR (and even a temporary extinction). While combustion efficiency is not affected (suggesting that flame poisoning is not observed) and char content is similar for all the phosphonate compounds, it must be assumed that flame retardancy is mainly driven by the delay to form a protective layer in the condensed phase. A fast formation and the expansion of the char are the keys to reaching the highest performances.

It would be interesting to apply this chemistry to different applications, such as PHU foams with FR properties.

Moreover, the influence of the oxidation state of phosphorus on the flame-retardant properties could be studied in other polymer matrices such as epoxy-acids for vitrimers synthesis.

## Figures and Tables

**Table 1:** DSC, Swelling rate and Gel content analyses of all the PHU thermosets.

Samples	T_g_ (°C) *	Swelling Rates (%)	Gel Content (%)
MBDAC	2	190	86
MBDAC-DOPO 1% P	21	300	92
MBDAC-DOPO 2% P	4	340	88
MBDAC-BPPO 1% P	33	220	84
MBDAC-BPPO 2% P	23	240	74
MBDAC DPP 1% P	24 and 63	200	90
MBDAC-DPP 2% P	−3 and 63	210	87
MBDAC-DEP 1% P	22	220	92
MBDAC-DEP 2% P	26	230	90

* T_g_ values determined by DSC (Midpoint-temperatures).

**Table 2:** Results of TGA analyses on materials.

Sample 1	T_d,5wt%_ (°C)	T_d,50wt%_ (°C)	Residue at 750 °C (%)
MBDAC	199	349	6
MBDAC-DOPO 1% P	200	349	11
MBDAC-DOPO 2% P	197	354	12
MBDAC-BPPO 1% P	198	348	15
MBDAC-BPPO 2% P	210	366	22
MBDAC DPP 1% P	189	340	18
MBDAC-DPP 2% P	196	340	23
MBDAC-DEP 1% P	188	340	11
MBDAC-DEP 2% P	201	340	20

**Table 3:** Main data from PCFC for all PHUs.

	pHRR (W/g)	TpHRR (°C)	THR (kJ/g)	Residue Fraction	ΔH (kJ/g)
MBDAC	157	403	19.3	0.01	19.6
MBDAC-DOPO 1%	169	379	18.9	0.06	20.1
MBDAC-DOPO 2%	162	366	18.9	0.09	20.8
MBDAC-BPPO 1%	138	352	17.7	0.09	19.3
MBDAC-BPPO 2%	159	334	19	0.15	22.3
MBDAC-DPP 1%	129	356	17.0	0.09	18.6
MBDAC-DPP 2%	142	340	18.4	0.10	20.4
MBDAC-DEP 1%	168	344	17.2	0.10	19.1
MBDAC-DEP 2%	171	337	16.2	0.13	18.7

**Table 4:** Main data from cone calorimeter tests for all PHUs.

	TTI (s)°	pHRR1 (kW·m^−2^)	TpHRR1 (s)	pHRR2 (kW·m^−2^)	TpHRR2 (s)
MBDAC	36	1644	90	/	/
MBDAC-DOPO 1%	32	1362	85	/	/
MBDAC-DOPO 2%	41	1134	95	/	/
MBDAC-BPPO 1%	37	1270	58	957	118
MBDAC-BPPO 2%	23	626	40	789	143
MBDAC-DPP 1%	32	629	55	1066	125
MBDAC-DPP 2%	48	509	68	1205	123
MBDAC-DEP 1%	28	427	38	922	155
MBDAC-DEP 2%	36	390	45	1065	165

**Table 5:** Main data from cone calorimeter tests for all PHUs.

	Residue Fraction	EHC (kJ·g−1)	X	THR (kJ·g−1)	TSR (m2·m−2)
MBDAC	0.02	20.1	1.03	19.8	820
MBDAC-DOPO 1%	0.09	20.1	1.00	18.4	1472
MBDAC-DOPO 2%	0.11	18.6	0.9	16.6	2320
MBDAC-BPPO 1%	0.09	19.9	1.03	18.1	1440
MBDAC-BPPO 2%	0.11	19.7	0.89	17.5	1976
MBDAC-DPP 1%	0.07	20	1.08	18.7	1475
MBDAC-DPP 2%	0.09	20.9	0.99	18.7	1585
MBDAC-DEP 1%	0.1	17.2	0.90	15.6	1180
MBDAC-DEP 2%	0.15	18.1	0.97	15.4	1163

**Table 6:** Functionalities of carbonate monomers.

	PPO DC	MBDAC	MBDAC-DOPO	MBDAC-BPPO	MBDAC-DEP	MBDAC-DPP
Functionality	2.0	4.0	3.0	3.0	3.0	3.0

## Data Availability

Not applicable.

## Supplementary Materials

The following supporting information can be downloaded at: https://www.mdpi.com/article/10.3390/molecules28020611/s1, Figure S1: Thiourea ^1^H NMR; Figure S2: PPO DC ^1^H NMR; Figure S3: MBDA ^1^H NMR in DMSO; Figure S4: MBDAC ^1^H NMR in DMSO; Figure S5: DOPO ^1^H NMR; Figure S6: DOPO ^31^P NMR; Figure S7: MBDA-DOPO ^1^H NMR; Figure S8: MBDA-DOPO ^31^P NMR; Figure S9: MBDAC-DOPO ^1^H NMR; Figure S10: MBDA-DOPO ^31^P NMR; Figure S11: BPPO ^1^H NMR; Figure S12: BPPO ^31^P NMR; Figure S13: MBDA-BPPO ^1^H NMR; Figure S14: MBDA-BPPO ^31^P NMR; Figure S15: MBDAC-BPPO ^1^H NMR; Figure S16: MBDAC-BPPO ^31^P NMR; Figure S17: DEP ^1^H NMR; Figure S18: DEP ^31^P NMR; Figure S19: MBDA-DEP ^1^H NMR; Figure S20: MBDA-DEP ^31^P NMR; Figure S21: MBDAC-DEP ^1^H NMR; Figure S22: MBDAC-DEP ^31^P NMR; Figure S23: DPP ^1^H NMR; Figure S24: DPP ^31^P NMR; Figure S25: MBDA-DPP ^1^H NMR; Figure S26: MBDA-DPP ^31^P NMR; Figure S27: MBDAC-DPP ^1^H NMR; Figure S28: MBDA-DPP ^31^P NMR; Figure S29: DSC thermogram of the MBDAC-DOPO thermosets; Figure S30: DSC thermogram of the MBDAC-BPPO thermosets; Figure S31: DSC thermogram of the MBDAC-DEP thermosets; Figure S32: DSC thermogram of the MBDAC-DPP thermosets. Table S1: Foams formulations with DOPO; Table S2: Foams formulations with BPPO; Table S3: Foams formulations with DEP; Table S4: Foams formulations with DPP. Video S1: Video_cone calorimeter_BPPO 1%P.

## Sample Availability

Samples of the compounds are available from the authors.
