# Supplementary material for: Influence of Phosphorus Structures and Their Oxidation States on Flame-Retardant Properties of Polyhydroxyurethanes
Source: Molecules. 2023 Jan 6;28(2):611. doi: 10.3390/molecules28020611 (PMC9867530; doi:10.3390/molecules28020611)
Supplement: Supplementary file 1 [file molecules-28-00611-s001.zip › molecules-2068341-supplementary.pdf]

Supplementary Materials

Influence of Phosphorus Structures and Their Oxidation States on Flame-Retardant Properties of Polyhydroxyurethanes.

Maxinne Denis [1], Guilhem Coste [1], Rodolphe Sonnier [2,*], Sylvain Caillol [1] and Claire Negrell [1,*]

[1] ICGM, Université de Montpellier, CNRS, ENSCM, Montpellier, 34000, France; maxinne.denis@enscm.fr (M.D.); guilhem.coste@enscm.fr (G.C.); sylcain.caillol@enscm.fr (S.C.)
[2] Polymers Composites and Hybrids (PCH), IMT Mines Ales, Ales, 30100, France
* Correspondence: rodolphe.sonnier@mines-ales.fr (R.S.); claire.negrell@enscm.fr (C.N.)

1. NMR spectra

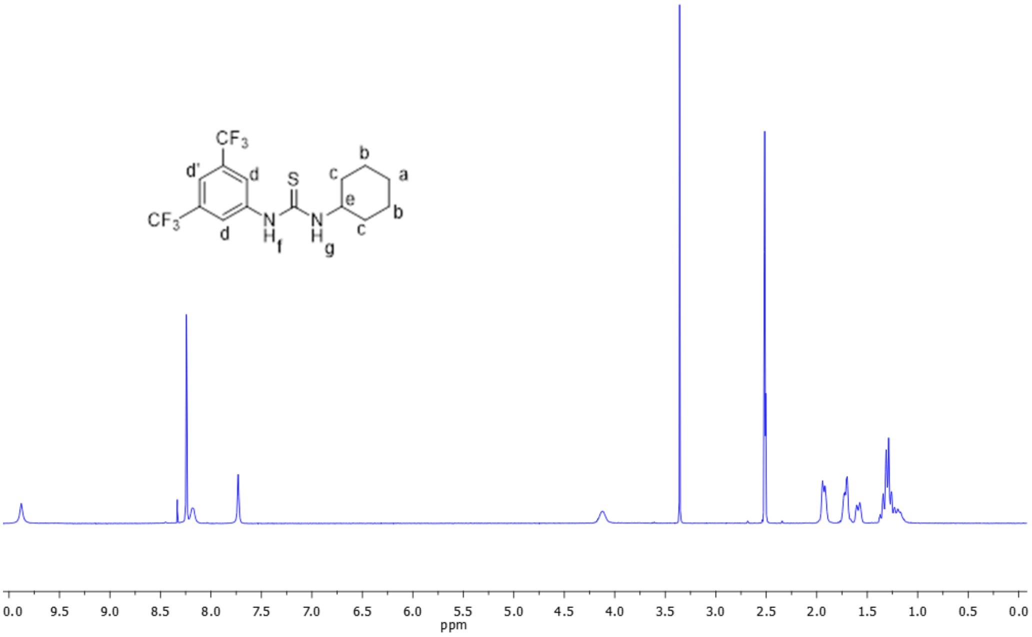

Figure 1: Thiourea ^{1}H NMR

^{1}H NMR (400 MHz, DMSO-d$_6$, ppm): δ = 1.44 (m, 4H, H$_b$), 1.5-1.99 (m, 4H, H$_c$), 2.66 (m, 3H, H$_e$ and H$_a$), 7.75 (s, 4H, H$_{d.}$), 8.18 (s, 1H, NH$_g$), 8.39 (s, 2H, H$_{d'}$), 9.89 (s, 1H, NH$_g$).

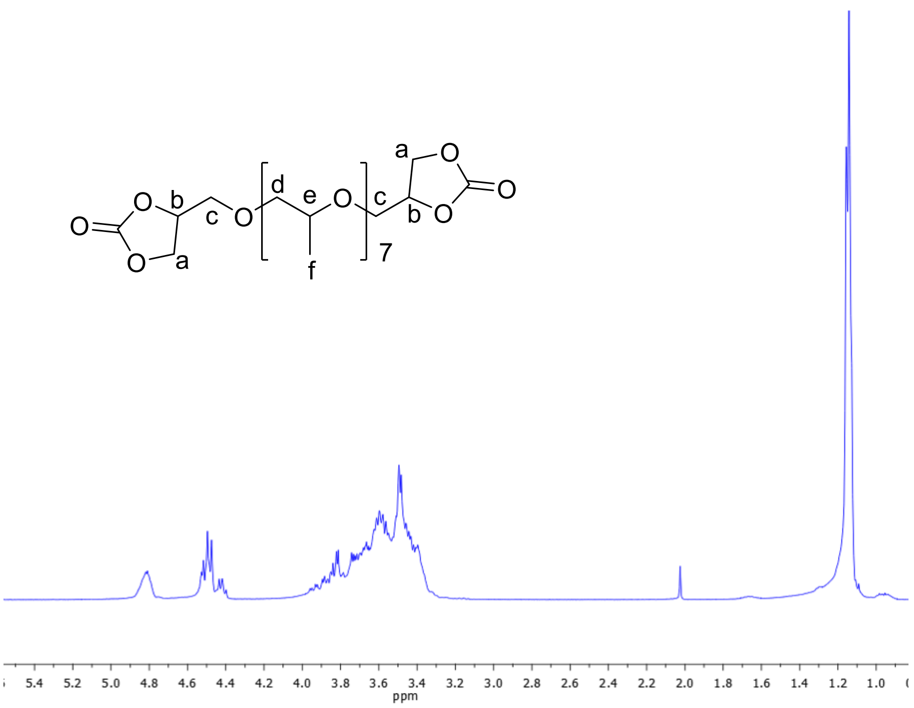

Figure 2: PPO DC ^{1}H NMR

^{1}H NMR (400 MHz, $CDCl_3$, ppm): δ = 1.15 (d, 21H, CH_3, H_f), 3.33-3.95 (m, 25H, H_c, H_d and H_e), 4.5 (m, 4H, CH_2, H_a), 4.8 (t, 2H, CH, H_b).

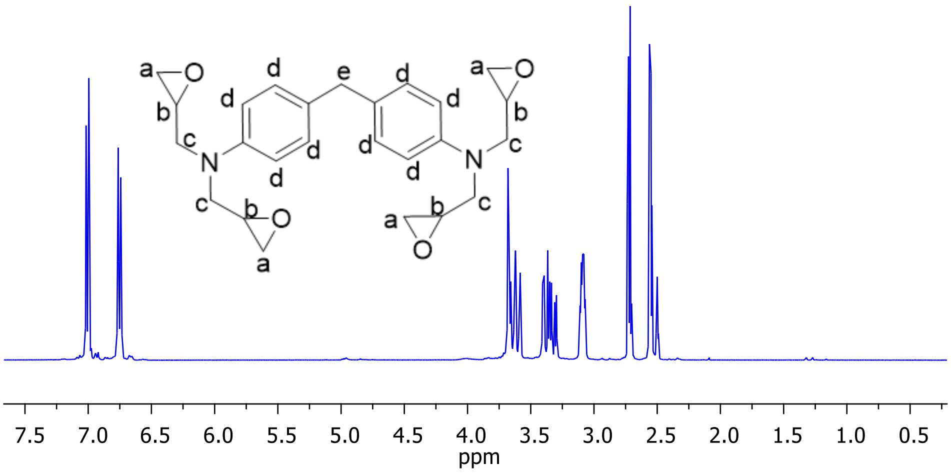

Figure 3: MBDA ^{1}H NMR

^{1}H NMR (400 MHz, DMSO-d_6, ppm): δ = 2.56 (m, 4H,H_a), 2.71 (m, 4H, H_a), 3.08 (m, 4H, H_b), 3.37 (m, 4H, H_c), 3.62 (dt, 4H, H_c), 3.68 (s, 2H, H_e), 6.68 (m, 4H, H_d), 6.99 (m, 4H, H_d).

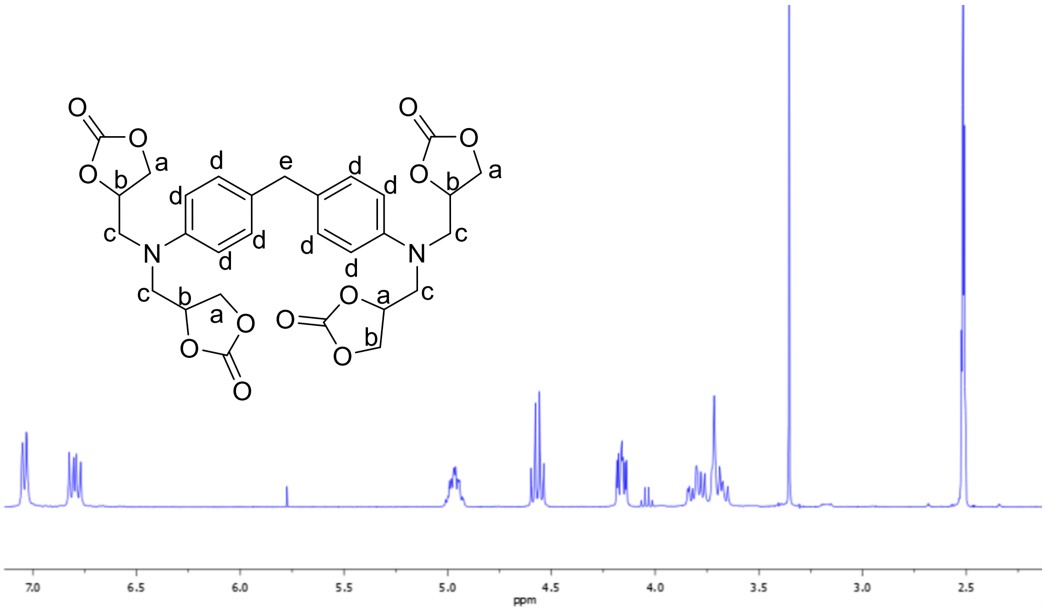

Figure 4: MBDAC ^{1}H NMR in DMSO

^{1}H NMR (400 MHz, DMSO-d6, ppm): δ = 3.92 (m, 10H, H_c and H_e), 4.27 (m, 4H, H_a), 4.51 (m, 4H, H_a), 4.89 (m, 4H, H_b), 6.72 (m, 4H, H_d), 7.10 (m, 4H, H_d).

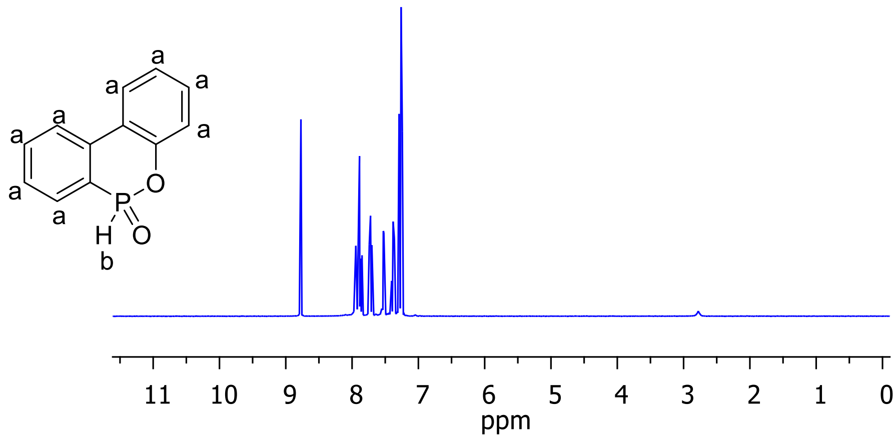

Figure 5: DOPO ^{1}H NMR

^{1}H NMR (400 MHz, $CDCl_3$, ppm): δ = 7.19-7.93 (m, 8H, H_a), 8.78 (s, 1H, H_b).

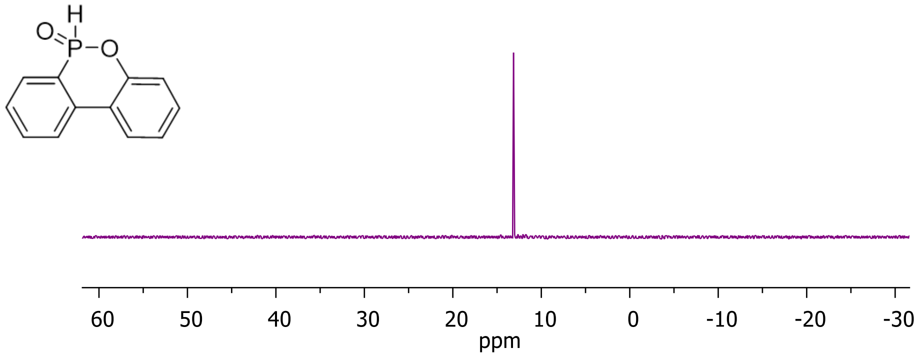

Figure 6: DOPO ^{31}P NMR

^{31}P NMR (400 MHz, $CDCl_3$, ppm): δ = 14.82 (P-H)

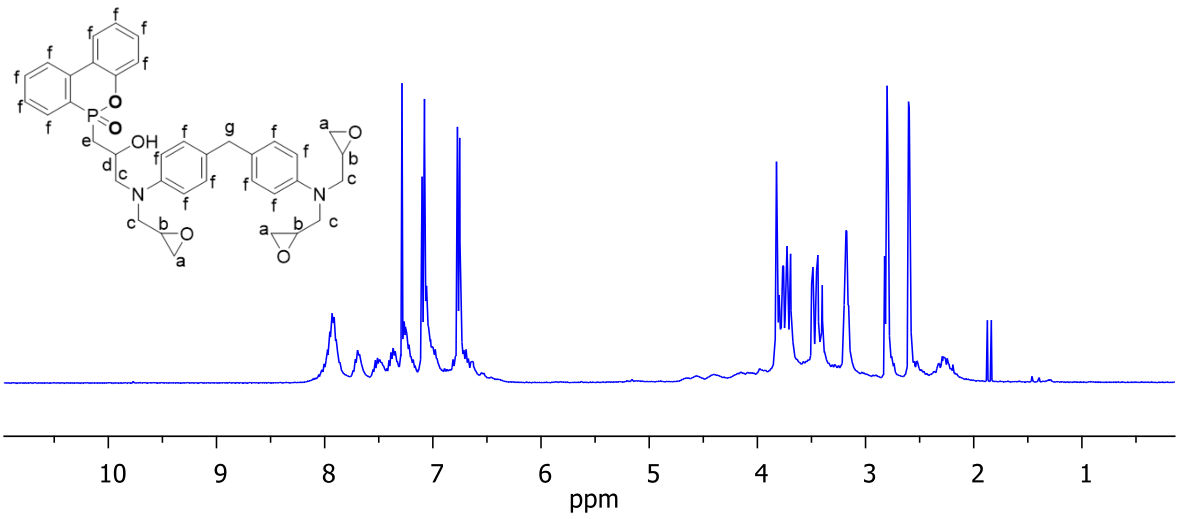

Figure 7: MBDA-DOPO ^{1}H NMR

^{1}H NMR (400 MHz, $CDCl_3$, ppm): δ = 2.27 (m, 2H, H_e), 2.61 (m, 3H, H_a), 2.79 (m, 3H, H_b), 3.17 (m, 3H, H_a), 3.45 (m, 4H, H_c), 3.70-3.80 (dt, 5H, H_c, H_d), 3.82 (s, 2H, H_g), 6.68-8.20 (m, 6H, H_f).

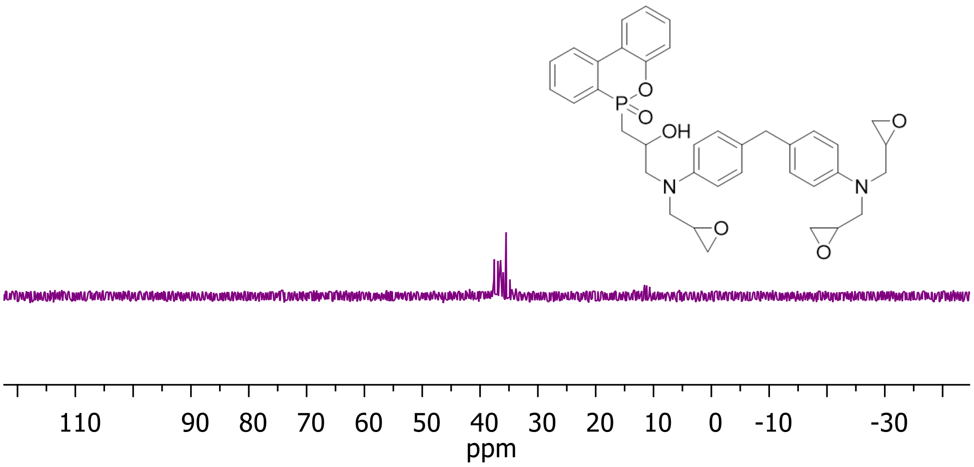

Figure 8: MBDA-DOPO ^{31}P NMR

^{31}P NMR (400 MHz, $CDCl_3$, ppm): δ = 36.04 (P-C)

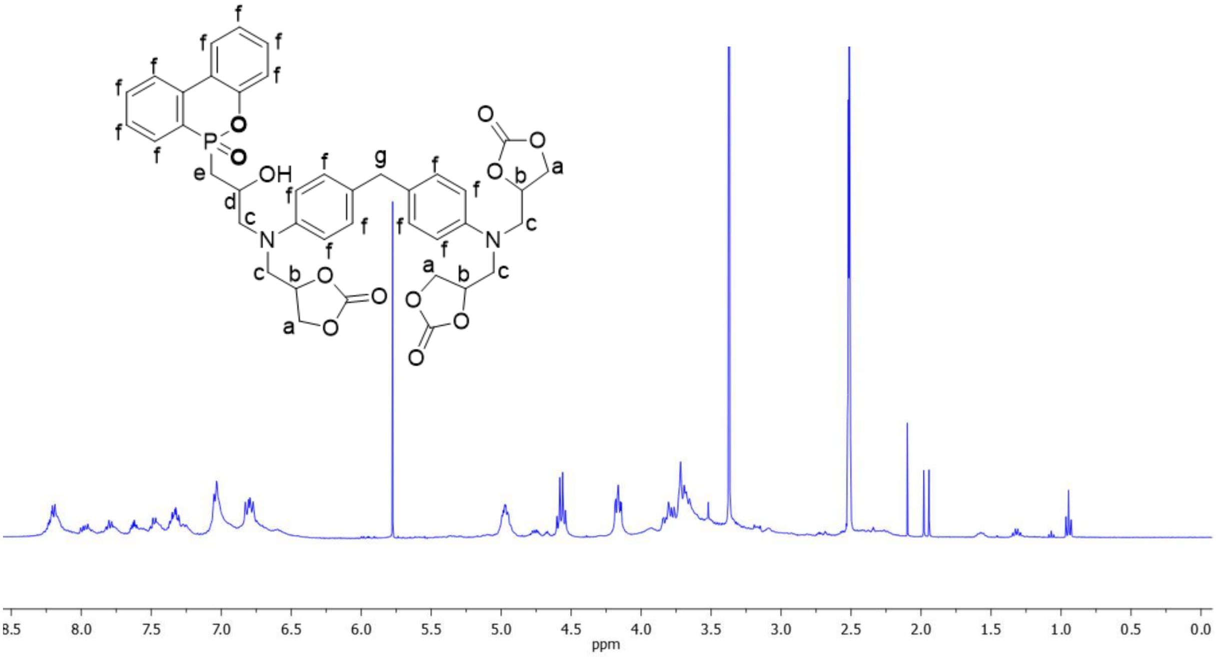

Figure 9: MBDAC-DOPO ^{1}H NMR

^{1}H NMR (400 MHz, DMSO-d_6, ppm): δ = 3.50-3.95 (m, 10H, H_e, H_C), 4.17 (m, 3H, H_a), 4.55 (m, 3H, CH_b), 4. 79 (m, 3H, H_a), 6.55-8.30 (m, 15H, H_f).

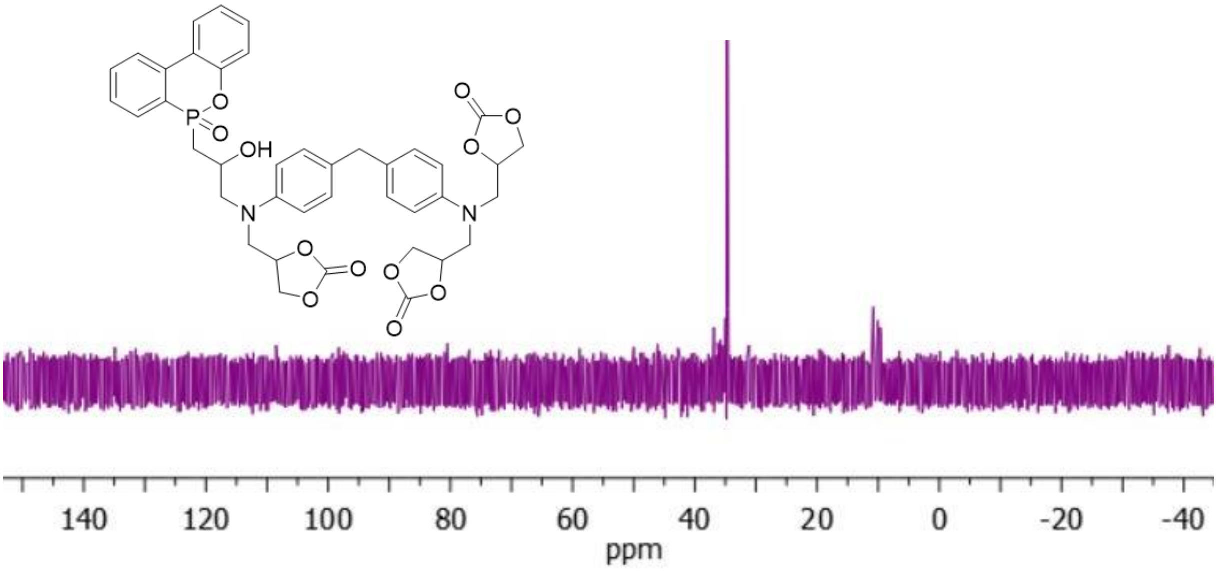

Figure 10: MBDA-DOPO ^{31}P NMR

^{31}P NMR (400 MHz, $CDCl_3$, ppm): δ = 35.95 (P-C)

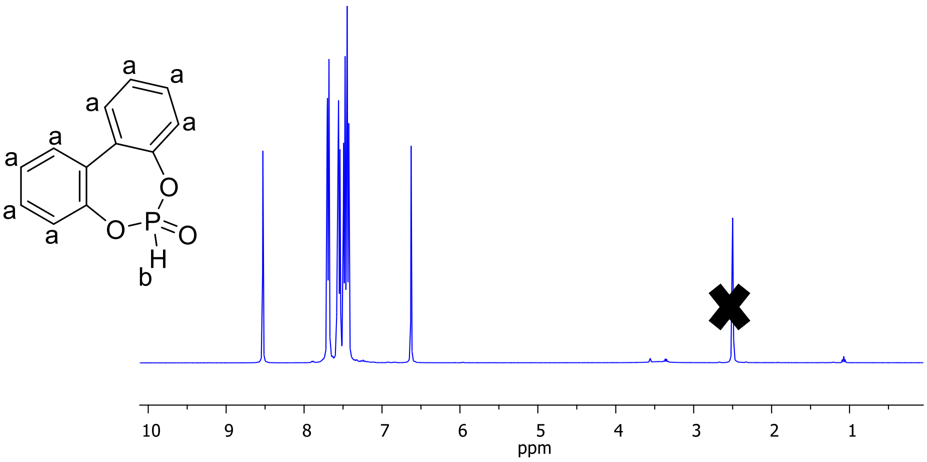

Figure 11: BPPO ^{1}H NMR

^{1}H NMR (400 MHz, DMSO-d_6, ppm): δ = 8.53 and 6.62 (s, H, H_b), 7.79-7.39 (m, 8H, H_a).

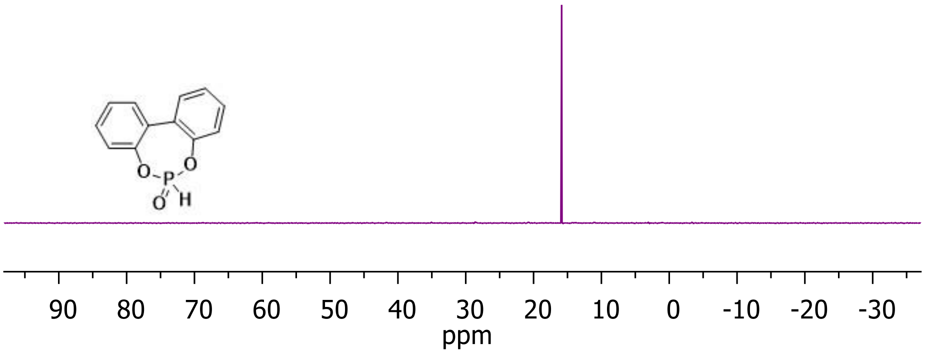

Figure 12: BPPO ^{31}P NMR

^{31}P NMR (400 MHz, DMSO-d_6, ppm): 15.9 ppm.

MBDA-BPPO

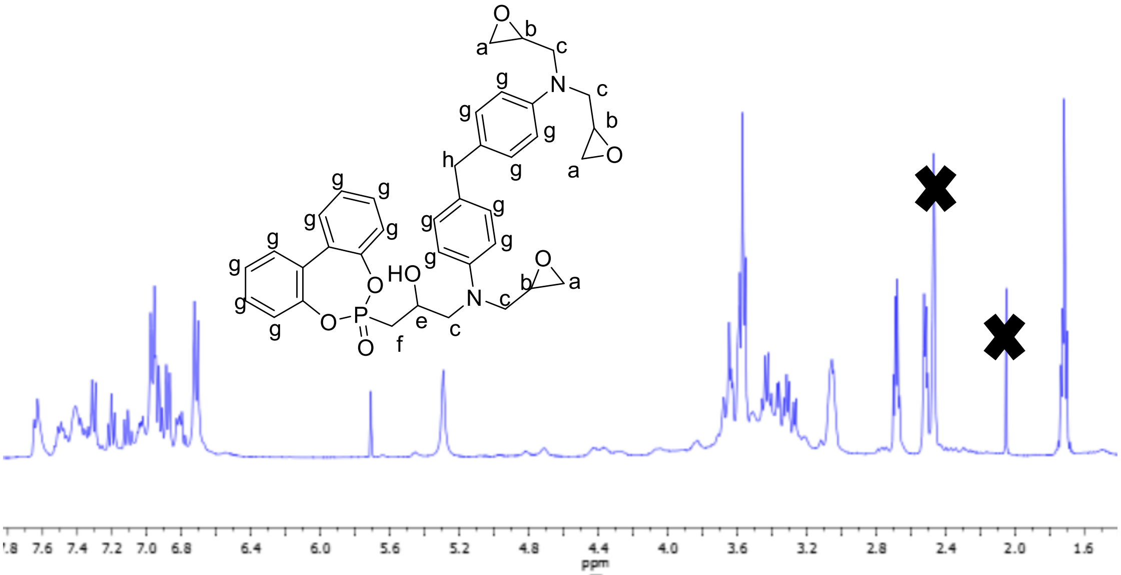

Figure 13: MBDA-BPPO ^{1}H NMR

^{1}H NMR (400 MHz, DMSO-d$_6$, ppm): δ = 1.15 (m, 2H, H_f), 2.42 (m, 3H, H_a), 2.52 (m, 3H, H_b), 2.68 (m, 3H, H_a), 3.2-3.6 (m, 8H,H_e, H_c, H_h), 6.6-7.7 (m, 15H, H_g).

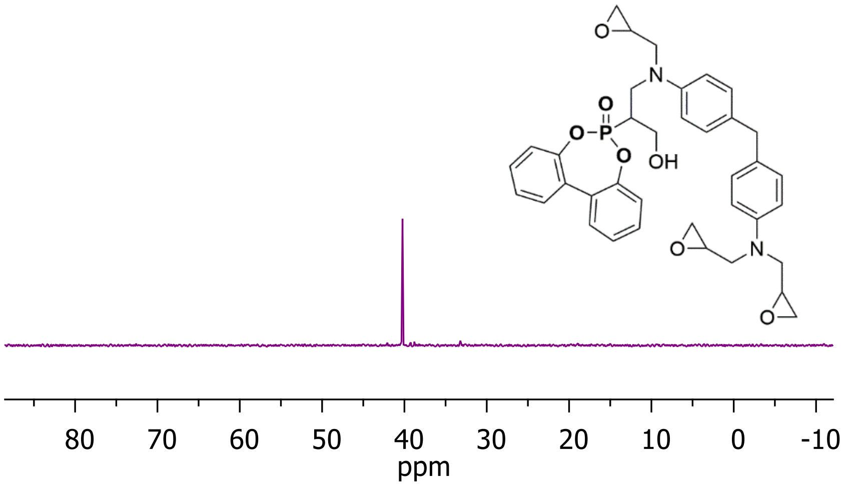

Figure 14: MBDA-BPPO ^{31}P NMR

^{31}P NMR (400 MHz, DMSO-d$_6$, ppm): 39.1 ppm.

MBDAC-BPPO

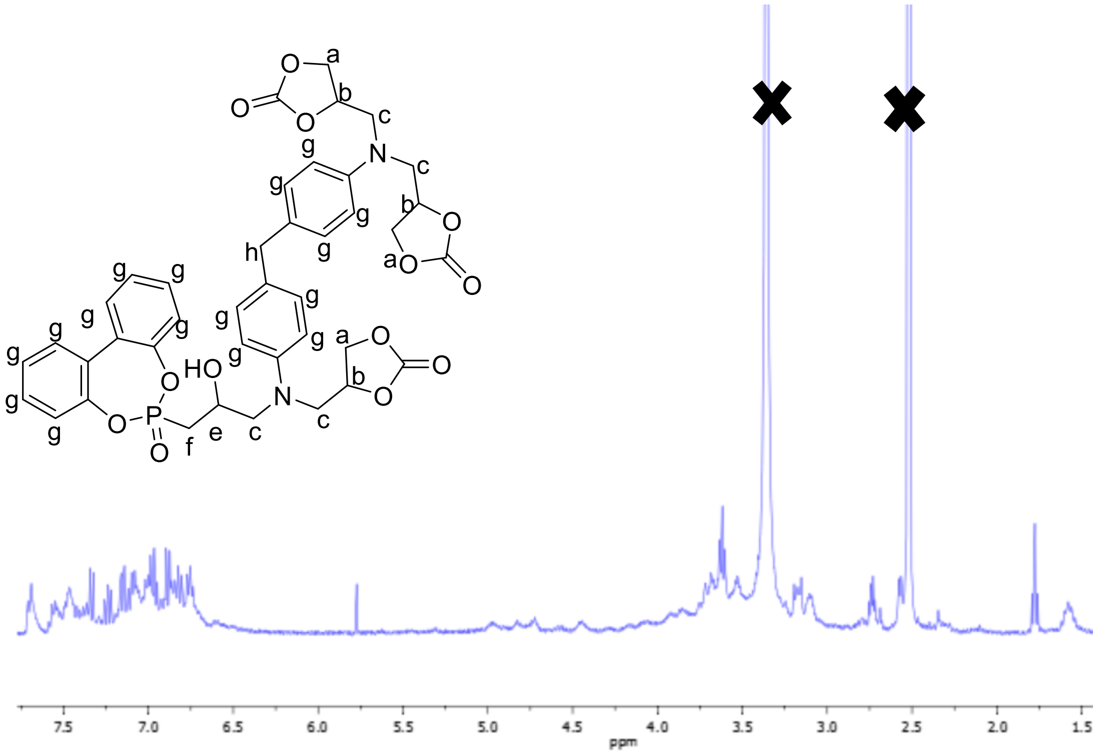

Figure 15: MBDAC-BPPO ^{1}H NMR

^{1}H NMR (400 MHz, DMSO-d$_6$, ppm): δ = 1.85 (m, 2H, H_f), 3.17-4.2 (m, 20H H_a, H_b, H_c, H_e, H_h), 6.55-7.75 (m, 16H, H_g).

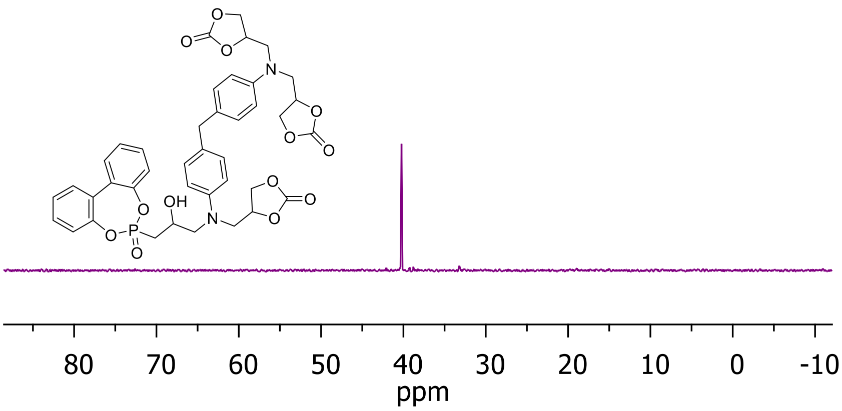

Figure 16: MBDAC-BPPO ^{31}P NMR

^{31}P NMR (400 MHz, DMSO-d_6, ppm): 40.02 ppm.
DEP

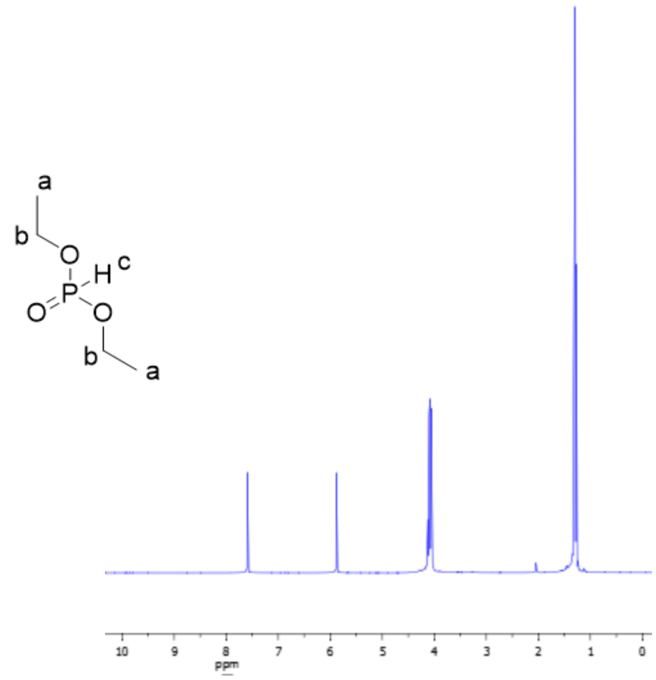

Figure 17: DEP ^{1}H NMR

^{1}H NMR (400 MHz, Acetone, ppm): δ = 1.31 (t, 6H, H_a), 4.03 (q, 4H H_b), 5.86 and 7.60 (a, 1H, H_c).

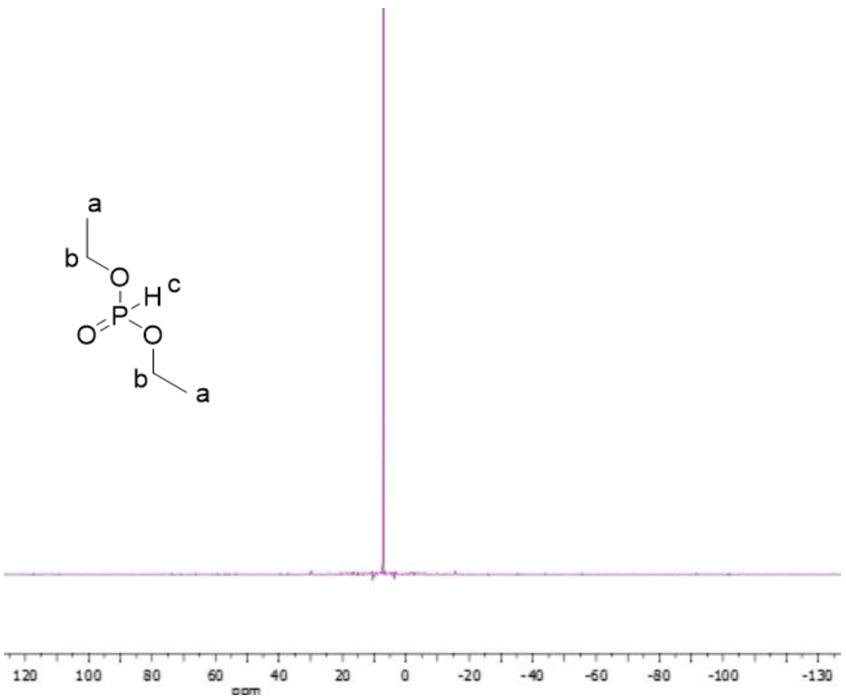

Figure 18: DEP^{31}P NMR

^{31}P NMR (400 MHz, DMSO-d$_6$, ppm): 6.63 ppm.

MBDA-DEP

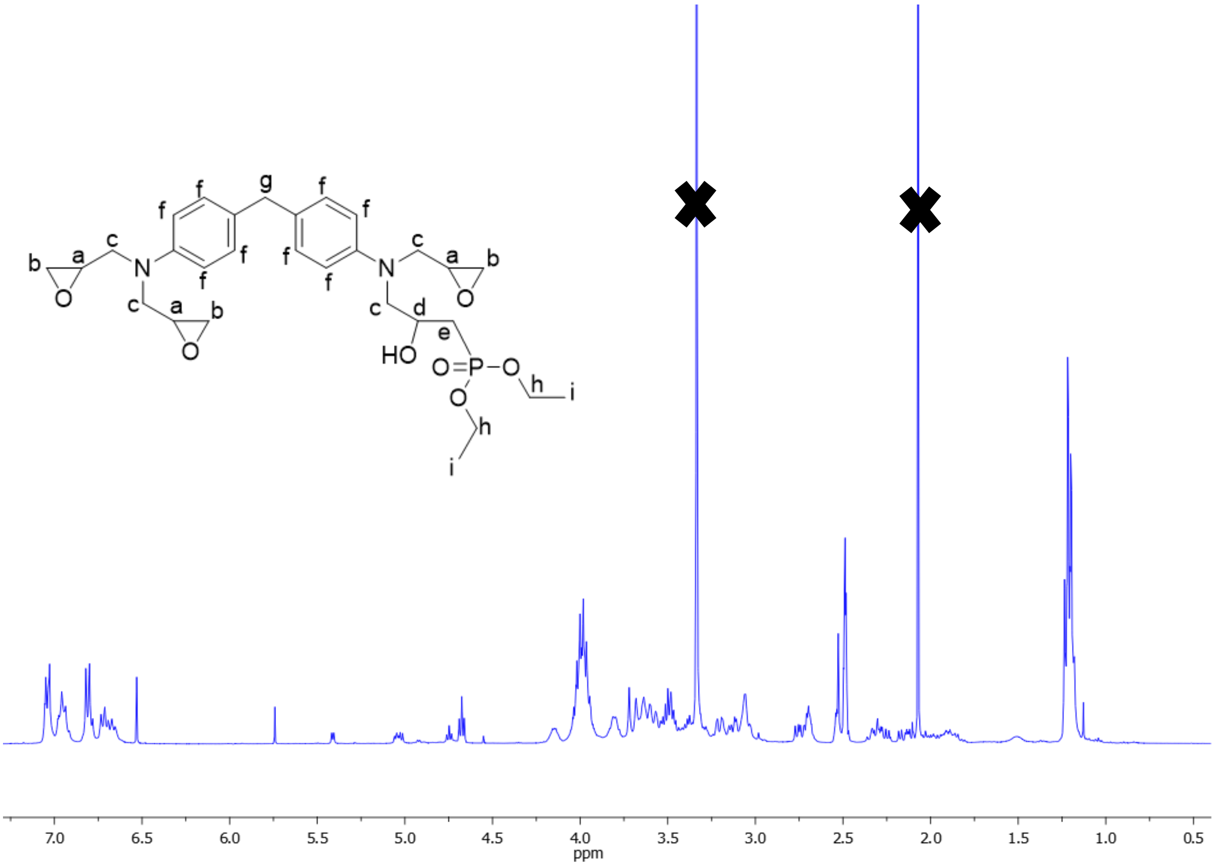

Figure 19: MBDA-DEP ^{1}H NMR

^{1}H NMR (400 MHz, DMSO-d_6, ppm): δ = 1.36 (m, 6H, H_i), 1.8-2.1 (m,2H, H_e), 2.45 (m, 3H, H_b), 2.53 (m, 3H, H_a), 2.68 (m, 3H, H_b), 3.10-3.72 (m, 7H, H_c, H_d), 3.82-4.19 (m, 6H, H_g, H_h), 6.76-7.15 (m, 8H, Har).

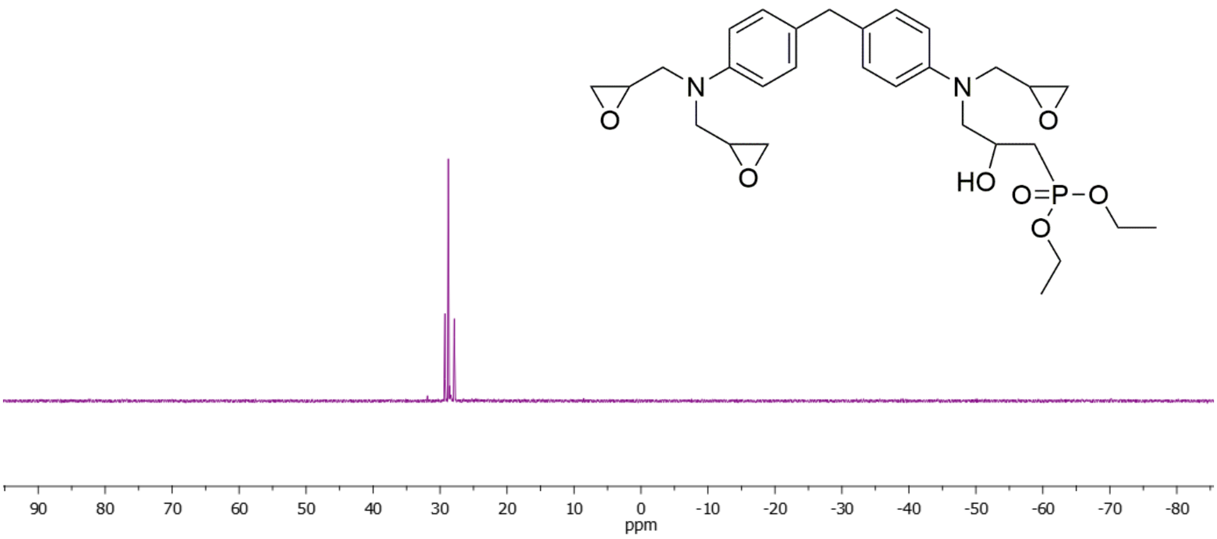

Figure 20: MBDA-DEP ^{31}P NMR

^{31}P NMR (400 MHz, DMSO-d_6, ppm): 29.6 ppm.

MBDAC-DEP

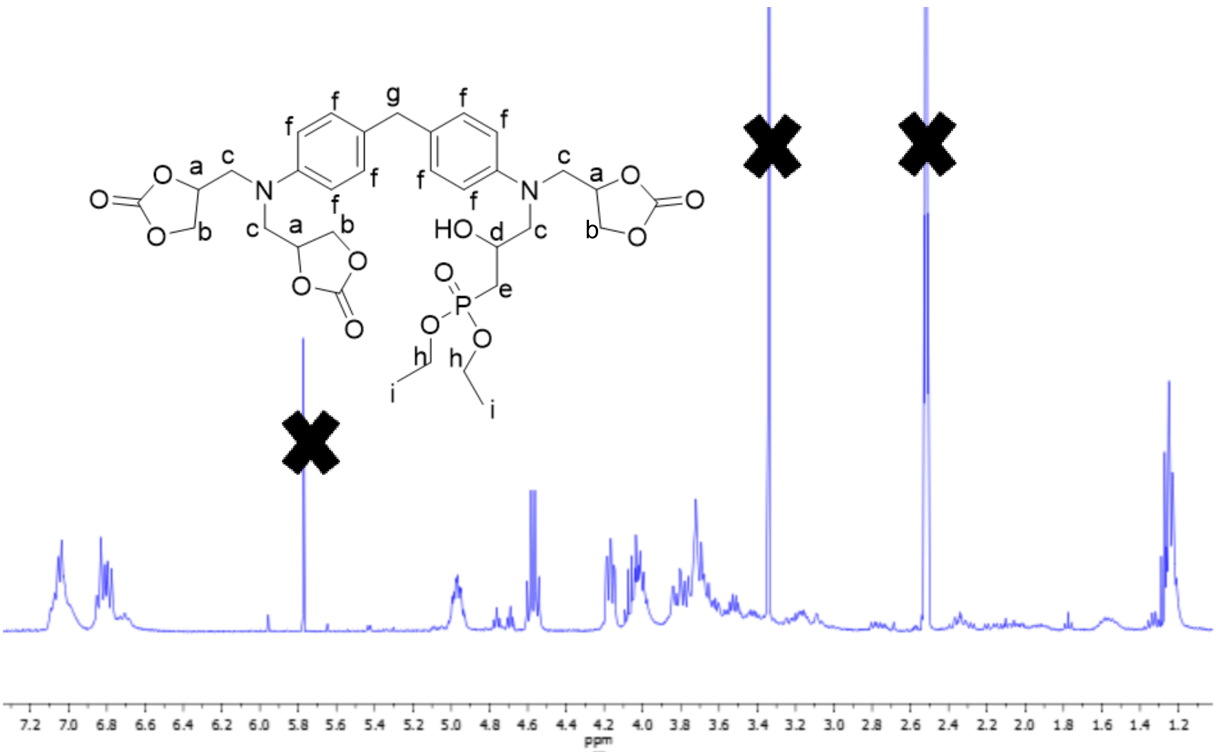

Figure 21: MBDAC-DEP ^{1}H NMR

^{1}H NMR (400 MHz, DMSO-d_6, ppm): δ = 1.25 (m, 6H, H_i), 2.34 (m (small), 2H, H_e), 3.4-3.82 (m, 5 ,H_c,H_d), 3.9-4.1 (m, 4H, H_a, H_g), 4.18 (m, 3H, H_b), 4.58 (q, 4H, H_h), 4.9 (m, 3H, H_a), 6.7-7.1 (m, 8H, H_f).

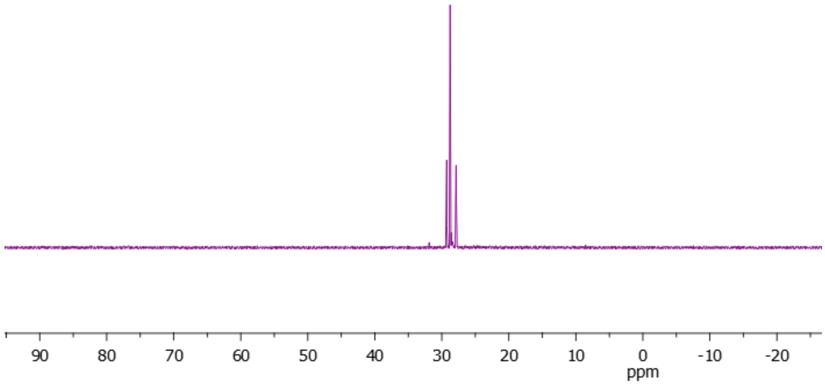

Figure 22: MBDAC-DEP ^{31}P NMR

^{31}P NMR (400 MHz, DMSO-d_6, ppm): 29.5 ppm.

DPP

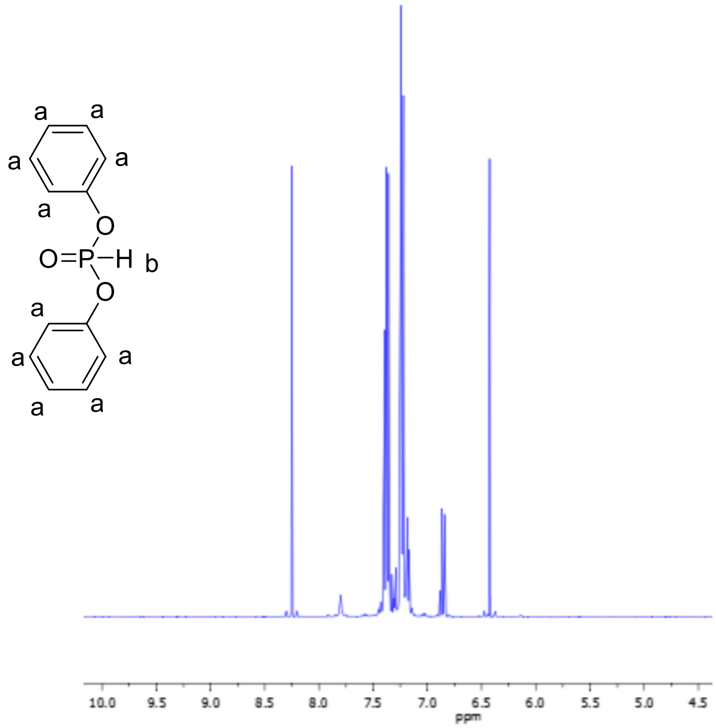

Figure 23: DPP ^{1}H NMR

^{1}H NMR (400 MHz, $CDCl_3$, ppm): δ = 6.42 and 8.24 (s, 1H, H_b), 6.86-7.45 (m, 10H, H_a).

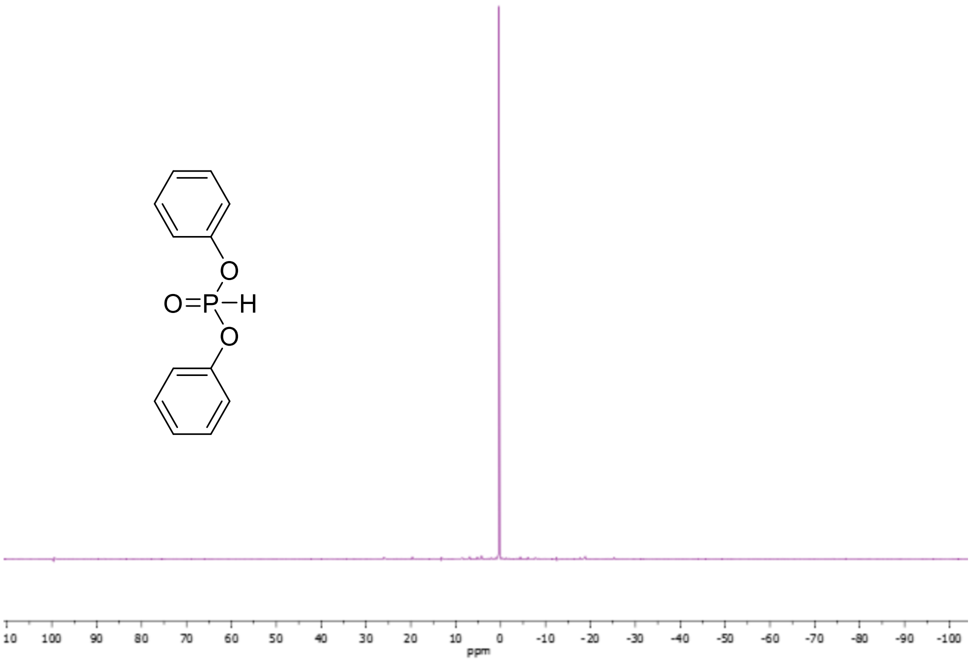

Figure 24: DPP ^{31}P NMR

^{31}P NMR (400 MHz, $CDCl_3$, ppm): 0.53 ppm.

MBDA-DPP

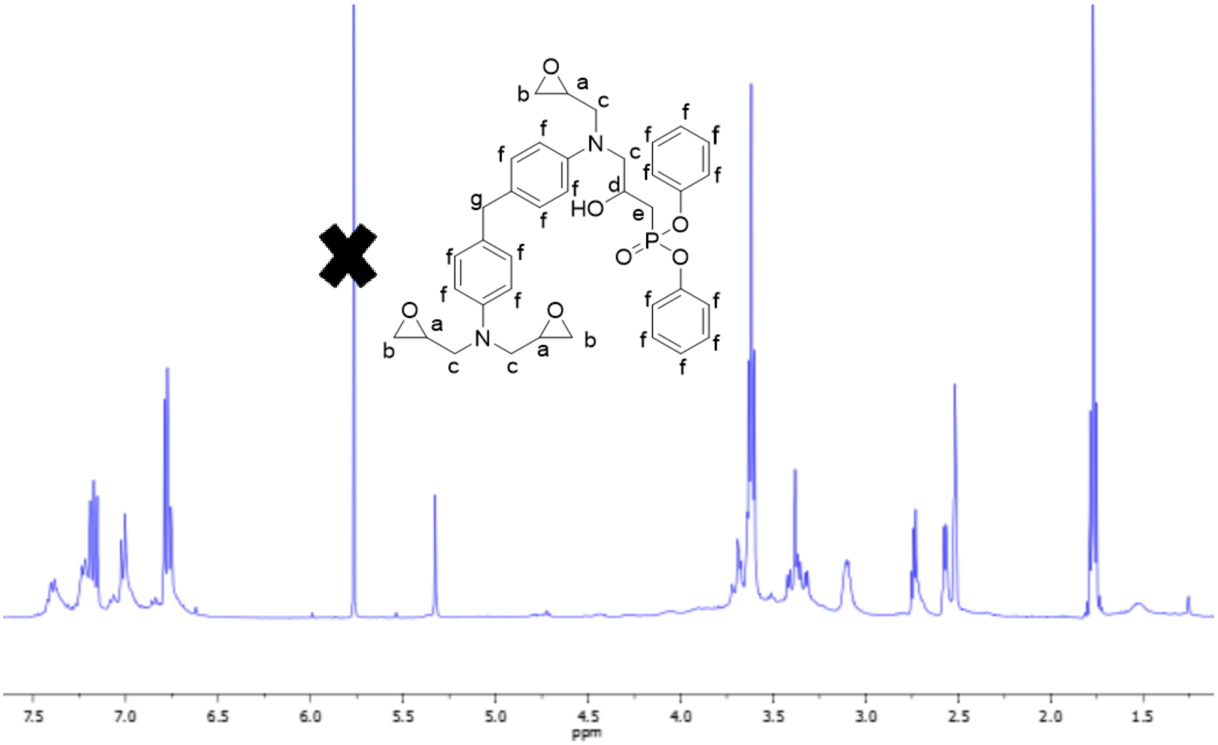

Figure 25: MBDA-DPP ^{1}H NMR

^{1}H NMR (400 MHz, DMSO-d_6, ppm): δ = 1.75 (m, 2H, H_e), 2.55 (m, 3H, H_a), 2.72 (m, 3H, H_b), 3.11 (m, 3H, H_a), 3.15 (m, 4H, H_c), 3.30-3.8 (m, 7H, H_c, H_g, H_d), 6.21-7.50 (m, 18H, H_f).

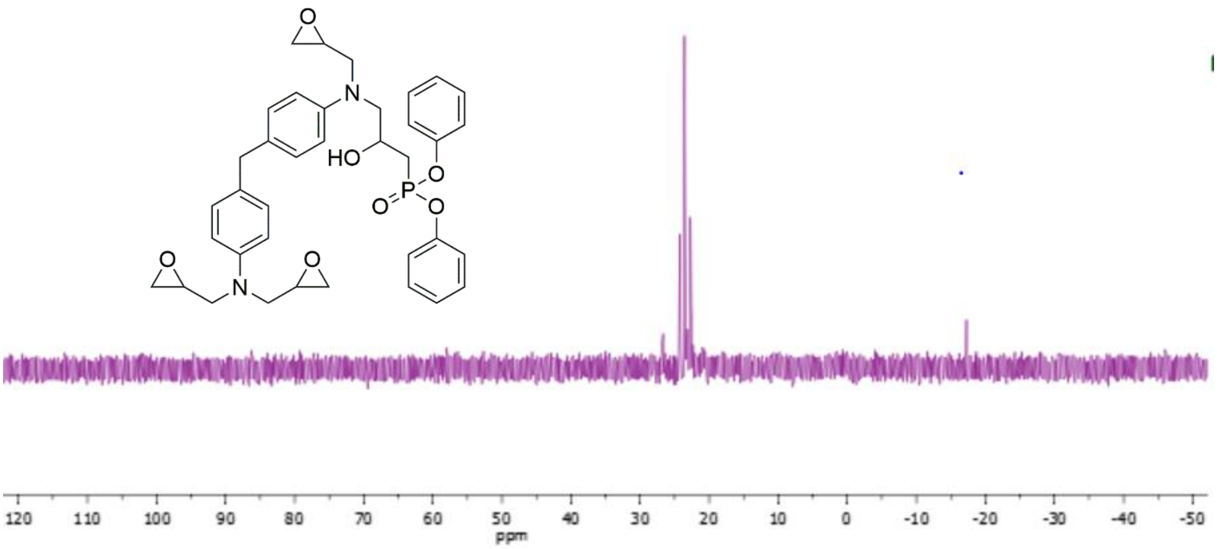

Figure 26: MBDA-DPP ^{31}P NMR

^{31}P NMR (400 MHz, DMSO-d_6, ppm): 23.4 ppm.

MBDAC-DPP

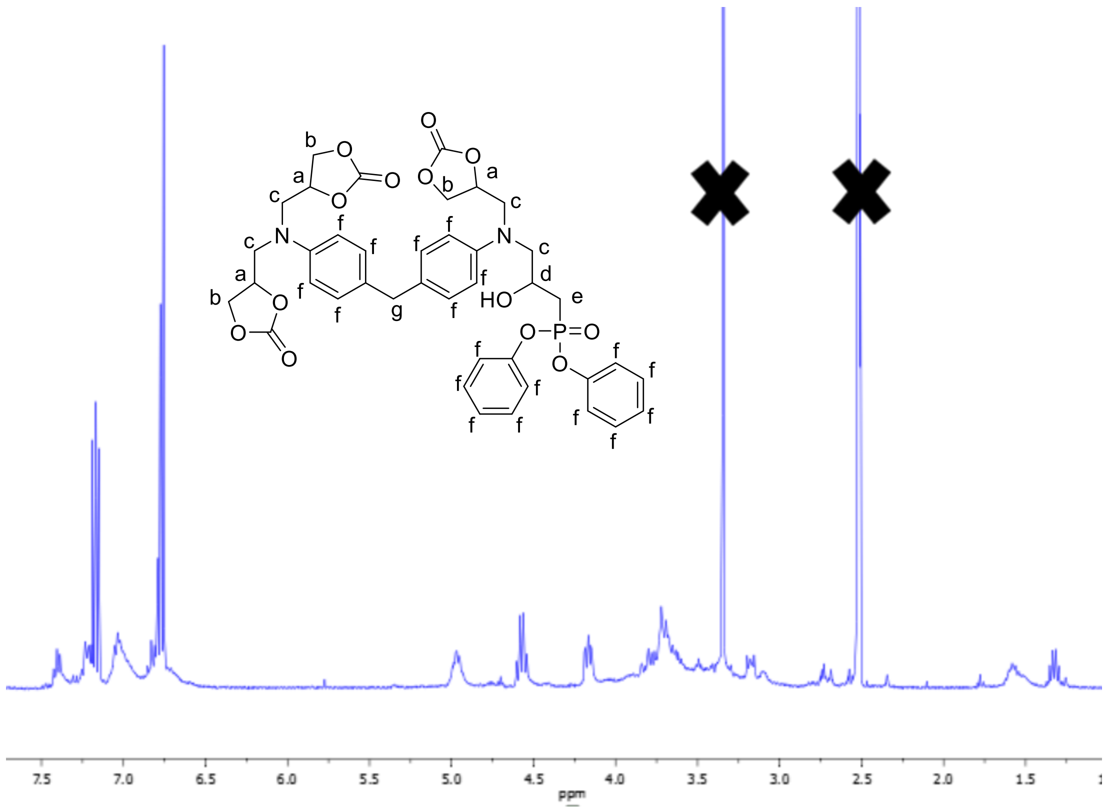

Figure 27: MBDAC-DPP ^{1}H NMR

^{1}H NMR (400 MHz, DMSO-d_6, ppm): δ = 1.6 (m, 2H, H_e), 3.25-3.80 (m, 9H ,H_c,, H_d), 4.2 (m, 3H, H_b), 4.6 (m, 3H, H_a), 4.92 (m, 3H, H_b), 6.57-7.47 (m, 18H, H_f).

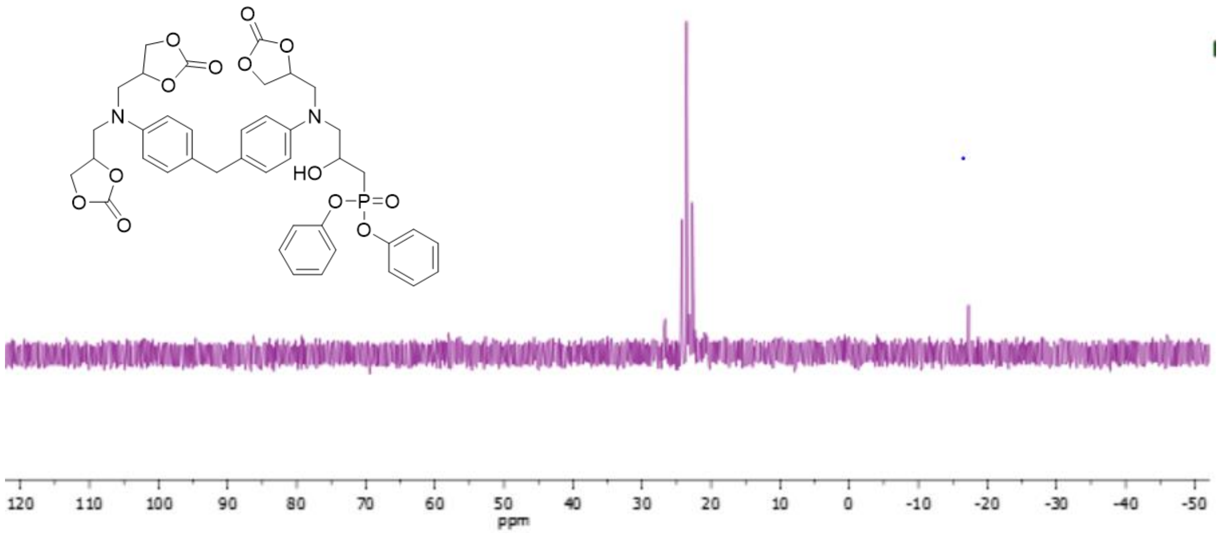

Figure 28: MBDA-DPP ^{31}P NMR

^{31}P NMR (400 MHz, DMSO-d_6, ppm): 23.4 ppm.

2. Foams formulations

Table 1: Foams formulations with DOPO

Foams	*PPOBC (g)*	*MBDAC (g)*	*MBDAC-DOPO (g)*	*EDR-148 (g)*	*Cat. (g)*
MBDAC ref	3.3	3.4	0	2.6	0.41
MBDAC-DOPO 1	3.3	2.0	2.5	2.3	0.41
MBDAC-DOPO 2	3.3	0	5.0	2.1	0.41

Table 2: Foams formulations with BPPO

Foams	*PPOBC (g)*	*MBDAC (g)*	*MBDAC-BPPO (g)*	*EDR-148 (g)*	*Cat. (g)*
MBDAC ref	3.3	3.4	0	2.6	0.41
MBDAC-BPPO 1	3.3	2.0	2.5	2.3	0.41
MBDAC-BPPO 2	3.3	0	5.0	2.1	0.41

Table 3: Foams formulations with DEP

Foams	*PPOBC (g)*	*MBDAC (g)*	*MBDAC-DEP (g)*	*EDR-148 (g)*	*Cat. (g)*
MBDAC ref	3.3	3.4	0	2.6	0.41
MBDAC-DEP 1	3.3	2.0	2.3	2.3	0.41

MBDAC-DEP 2	3.3	0.3	4.8	2.1	0.41

Table 4: Foams formulations with DPP

Foams	*PPOBC (g)*	*MBDAC (g)*	*MBDAC-DPP (g)*	*EDR-148 (g)*	*Cat. (g)*
MBDAC ref	3.3	3.4	0	2.6	0.41
MBDAC-DPP 1	3.3	2.0	2.5	2.3	0.41
MBDAC-DPP 2	3.3	0	5.0	2.1	0.41

3. Differential Scanning Calorimetry

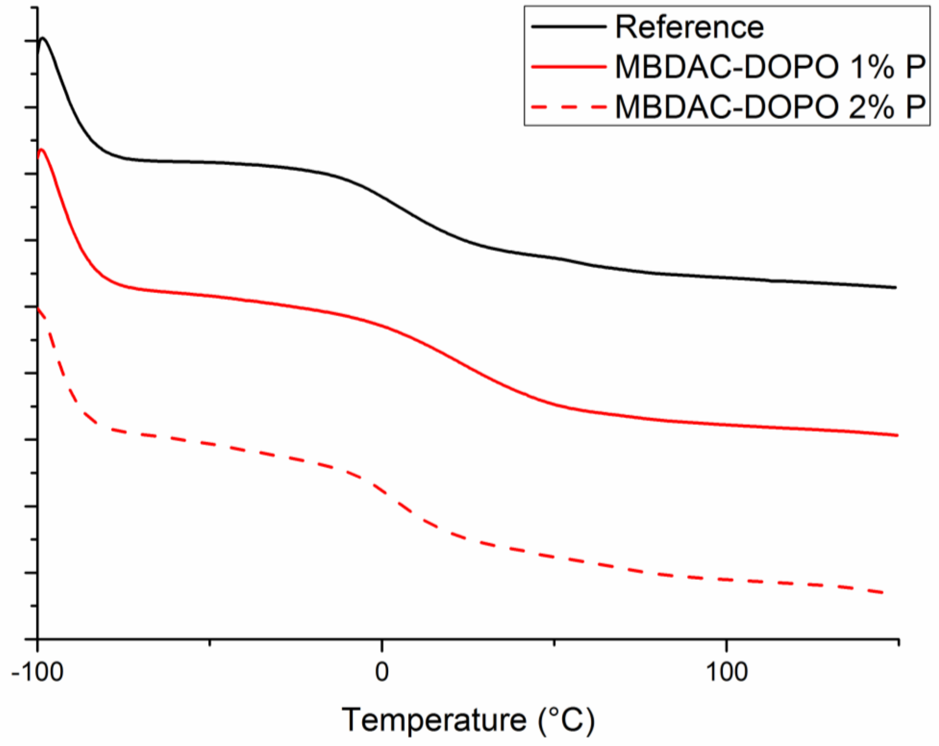

Figure 29: DSC thermograms of the MBDAC-DOPO thermosets

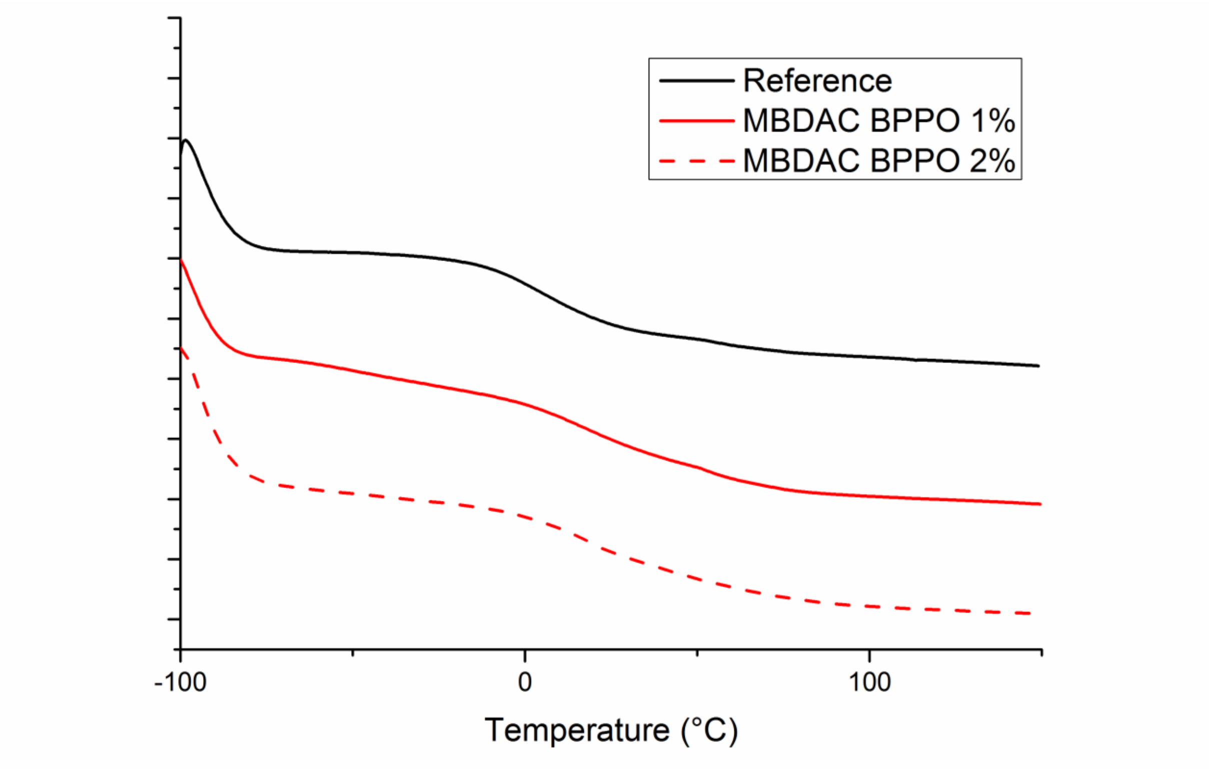

Figure 30: DSC thermograms of the MBDAC-BPPO thermosets

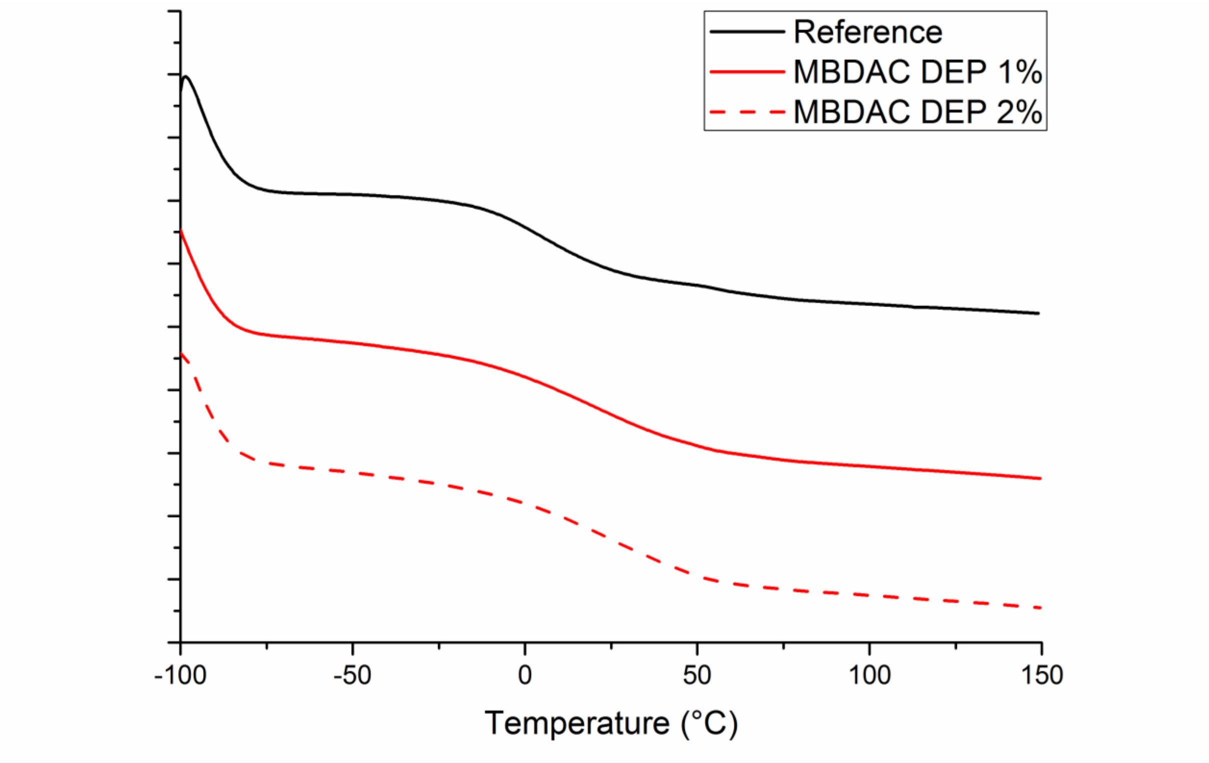

Figure 31: DSC thermograms of the MBDAC-DEP thermosets

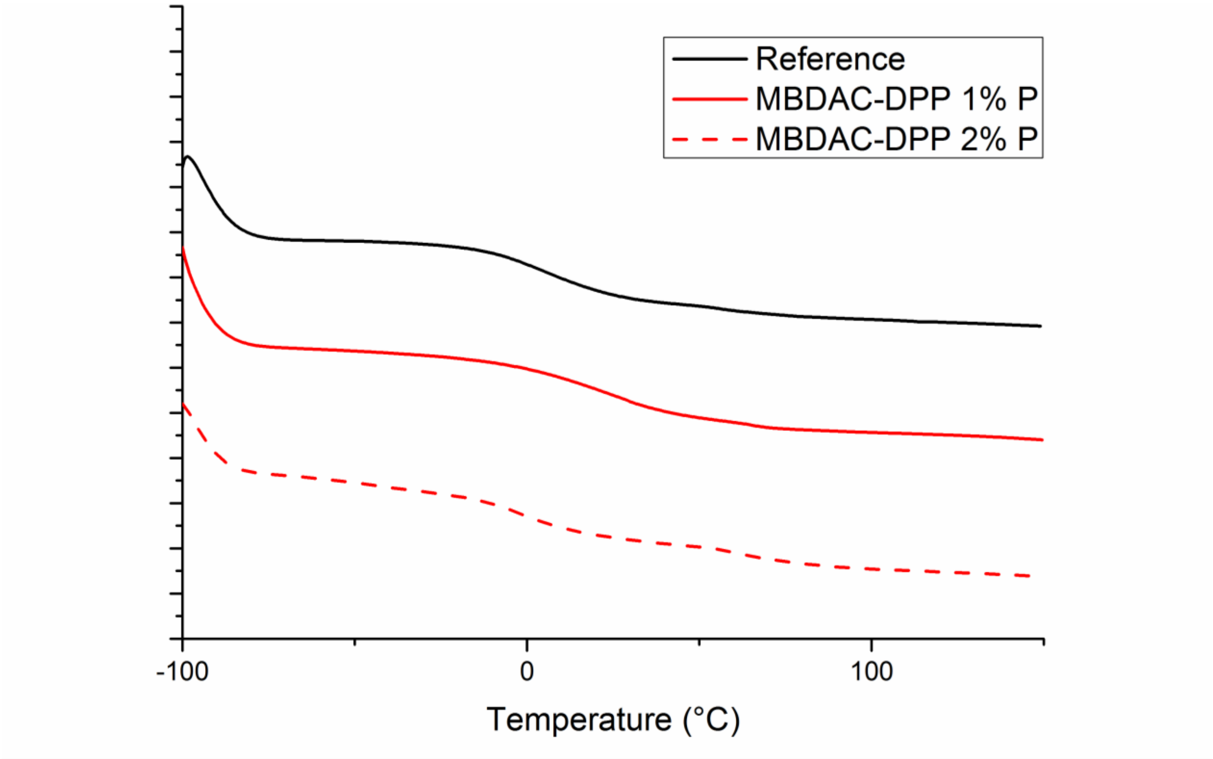

Figure 32: DSC thermograms of the MBDAC-DPP thermosets
